# Identification of a Fusobacterial RNA-binding protein involved in host small RNA-mediated growth inhibition

**DOI:** 10.1038/s41368-025-00378-4

**Published:** 2025-06-11

**Authors:** Pu-Ting Dong, Mengdi Yang, Jie Hu, Lujia Cen, Peng Zhou, Difei Xu, Peng Xiong, Jiahe Li, Xuesong He

**Affiliations:** 1 Department of Microbiology, The American Dental Association Forsyth Institute, Cambridge, MA USA; 2 Department of Bioengineering, Northeastern University, Boston, MA USA; 3 University of Science and Technology of China, Hefei, China; 4 Department of Biomedical Engineering, Suzhou Institute for Advanced Study, University of Science and Technology of China, Suzhou, China; 5 Department of Microbiology and Molecular Genetics, University of Texas McGovern Medical School, Houston, TX USA; 6 Department of Biomedical Engineering, College of Engineering and School of Medicine, University of Michigan, Ann Arbor, MI USA

**Keywords:** Cellular microbiology, Molecular medicine

## Abstract

Host-derived small RNAs are emerging as critical regulators in the dynamic interactions between host tissues and the microbiome, with implications for microbial pathogenesis and host defense. Among these, transfer RNA-derived small RNAs (tsRNAs) have garnered attention for their roles in modulating microbial behavior. However, the bacterial factors mediating tsRNA interaction and functionality remain poorly understood. In this study, using RNA affinity pull-down assay in combination with mass spectrometry, we identified a putative membrane-bound protein, annotated as P-type ATPase transporter (PtaT) in *Fusobacterium nucleatum* (*Fn*), which binds *Fn*-targeting tsRNAs in a sequence-specific manner. Through targeted mutagenesis and phenotypic characterization, we showed that in both the *Fn* type strain and a clinical tumor isolate, deletion of *ptaT* led to reduced tsRNA intake and enhanced resistance to tsRNA-induced growth inhibition. Global RNA sequencing and label-free Raman spectroscopy revealed the phenotypic differences between *Fn* wild type and PtaT-deficient mutant, highlighting the functional significance of PtaT in purine and pyrimidine metabolism. Furthermore, AlphaFold 3 prediction provides evidence supporting the specific binding between PtaT and *Fn*-targeting tsRNA. By uncovering the first RNA-binding protein in *Fn* implicated in growth modulation through interactions with host-derived small RNAs (sRNAs), our study offers new insights into sRNA-mediated host-pathogen interplay within the context of microbiome-host interactions.

## Introduction

Human mucosal surfaces provide a first line of protection against infectious bacteria through a complex array of innate and adaptive immunity.^1,2^ The symbiotic relationship with hundreds of microbial species requires a finely tuned response at the mucosal surface to prevent the overgrowth of opportunistic pathogens while sparing the beneficial microbes.^3–5^ Recent studies have highlighted certain processes, including host-derived small RNAs (sRNAs), that contribute to maintaining host-microbial homeostasis.^6,7^ The intricate interplay between host-derived sRNAs and host-associated microbiome has emerged as a fascinating area of investigation, offering profound implications for understanding host-microbe interactions.^8–11^

Of particular interest are transfer RNA-derived small RNAs (tsRNAs) produced by endonucleases following the splicing of precursor or mature tRNAs.^12^ tsRNAs have been shown to carry out important biological functions, such as epigenetic regulation, cell-cell communication, stress response and regulation of gene expression,^12–16^ and can be aberrantly expressed in several disease conditions.^12,17^ Increasing lines of evidence also indicate that tsRNAs may play an important role in host-pathogen interactions.^17^ Among human pathobionts, *Fusobacterium nucleatum* (*Fn*) represents a key player in various human diseases,^18–21^ ranging from periodontitis^22,23^ to colorectal cancer.^19,24,25^ Previous studies demonstrated that an immortalized human oral keratinocyte cell line releases two exosome-borne tsRNAs, tsRNA-000794 and tsRNA-020498, when challenged with *Fn*, and these tsRNAs exhibit highly selective, *Fn*-targeting antimicrobial activity via their ribosome-targeting functions.^26^ Further transcriptomic analysis indicated that these host-derived tsRNAs may also interfere with other cellular functions, such as purine synthesis and hemin uptake, to inhibit the growth of *Fn*.^26^ Chemical modification of these *Fn*-targeting tsRNAs, termed MOD-tsRNAs,^26^ led to enhanced potency (over three orders of magnitude) while maintaining specificity,^26^ thus offering promising prospects for targeted antimicrobial strategies.

In this study, we aimed to further elucidate bacterial genetic determinants involved in tsRNA-mediated growth inhibition. By employing affinity pull-down, targeted mutagenesis and bacteria phenotypic characterization, coupled with protein–RNA interaction prediction, we identified in *Fn* a putative P-type ATPase as a binding protein for host-derived small RNAs and involved in mediating tsRNA-induced growth inhibition.

## Results

### Identification of a putative membrane protein binding to *Fn*-targeting tsRNAs

In previous studies,^16,26^ we identified two host-derived *Fn*-targeting tsRNAs, tsRNA-000794 and tsRNA-020498, which are produced by an immortalized human oral keratinocyte cell line when challenged with *Fn*. These tsRNAs exhibit antimicrobial activity against *Fn* with high specificity via their ribosome-targeting functions.^26^

**Fig. 1:**
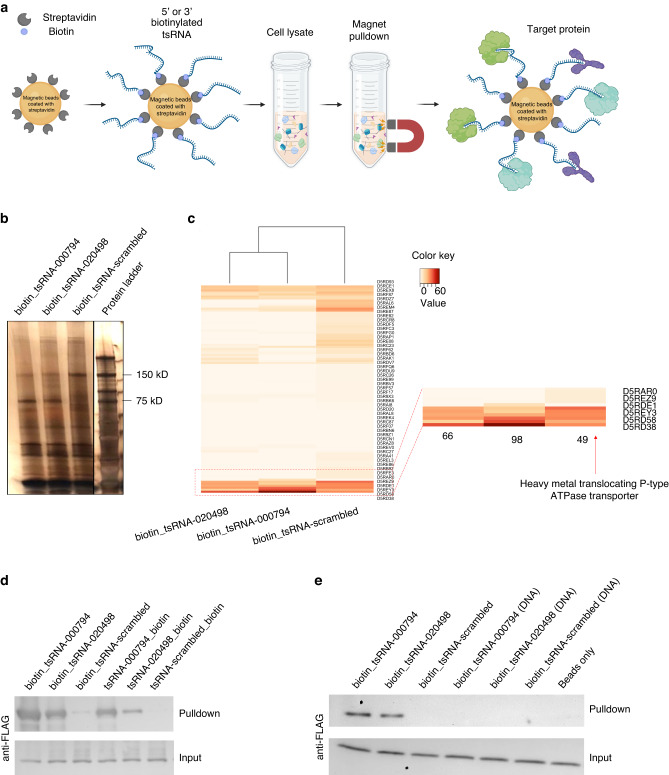
Identification of a putative tsRNA-binding protein in *Fn*. **a** Illustration of the workflow for an RNA affinity pulldown assay to identify RNA-binding proteins. 5′ or 3′ biotinylated RNA oligonucleotides are immobilized on the surface of streptavidin-conjugated paramagnetic microparticles to capture RNA-interacting proteins from cell lysate. **b** Silver staining of proteins from the biotinylated RNA pulldown in *Fn* ATCC 23726. Results are representative images of four independent experiments. **c** Heatmap showing the relative abundance of proteins isolated from the gel band at 75 kDa through mass spectrometry. The most significant band labeled as ‘D5RD38’ (P-type ATPase transporter, PtaT) was highlighted by a red arrow. **d** Validating the interaction between biotinylated tsRNA and recombinant PtaT. 5′ or 3′ biotin tsRNA-mediated affinity pulldown was performed using the total lysate of *E. coli* BL21 Rosetta, in which FLAG-tagged PtaT was recombinantly expressed. 5′ and 3′ biotinylated scrambled RNA serves as a negative control. The lower panel (input) represents an equal amount of total lysates used, and the upper panel (pulldown) indicates the amount of FLAG-tagged PtaT specifically interacting with different biotinylated RNA. A representative image of two independent experiments is shown. **e** Purified recombinant FLAG-PtaT can specifically bind tsRNA-000794 and tsRNA-020498 but not their DNA counterparts. Recombinant FLAG-tagged PtaT, which was purified from *E. coli* BL21 Rosetta, was directly used for the pulldown assay with 5′ biotinylated tsRNA. 5′ biotinylated scrambled RNA and beads only served as the negative control

To further elucidate genetic determinants and identify additional targets in *Fn* for tsRNA-mediated growth inhibition, we performed RNA affinity pulldown assay,^27^ an established method for identifying sRNA-associated proteins in mammalian cells. Specifically, we prepared total bacterial lysate containing both cytoplasmic and membrane fractions, and used synthetic biotinylated tsRNA and streptavidin-conjugated magnetic beads to pull down putative proteins from the lysate that interact strongly with tsRNA-000794 but less so with the scrambled control, which could in principle enrich for target proteins (Fig. 1a). As shown in the silver staining (Fig. 1b), a band with a molecular weight of ~75 kDa was enriched in the samples from biotinylated tsRNA-000794 but noticeably less from the biotinylated scrambled RNA in three tested *Fn* strains (*Fn* ATCC 23726, *Fn* ATCC 25586 and *Fn* ATCC 10953) (SI Fig. S1a). Additionally, gel bands with the same molecular weight were detected in the tsRNA pulldown assays using six *Fn* clinical tumor isolates (CTIs) (SI Fig. S1b). In comparison, when the same RNA affinity pulldown assay was applied in total cell lysates from *Streptococcus mitis* (*Sm*) and *Porphyromonas gingivalis* (*Pg*), biotinylated tsRNA-000794 or tsRNA-020498 failed to enrich for any specific band compared to that of biotinylated scrambled RNA (SI Fig. S1c). Taken together, the presence of a unique protein band pulled down by two different *Fn*-targeting tsRNAs from the lysates of *Fn* but not *Sm* or *Pg* suggests specificity in their interaction. This finding supports our previous observations of species- and sequence-specific growth inhibition of tsRNA-000794 and tsRNA-020498 against *Fn*.^26^

We then sought to identify the proteins specifically pulled down by the *Fn*-targeting tsRNAs via mass spectrometry. While certain RNases or known RNA-binding proteins were found in the gel bands at ~75 kDa of molecular weight, they were not specifically enriched by the two *Fn*-targeting tsRNAs when compared to the scrambled control RNA (Fig. 1c). In contrast, a putative membrane-bound P-type ATPase transporter (PtaT) was specifically associated with biotinylated tsRNA-000794 and tsRNA-020498 in *Fn* ATCC 23726, *Fn* ATCC 25586 and *Fn* ATCC 10953 as well as six *Fn* CTIs. By aligning the amino acid sequences of PtaT, we found that ATCC 23726 and CTI-2 share identical protein sequences for PtaT, while other strains share 96–99% identities with ATCC 23726 (Supplementary Table S1). The conserved sequence for PtaT suggests that this protein may play a common role in tsRNA-mediated growth inhibition in the tested *Fn* strains. However, since bioinformatic prediction suggested the function of PtaT in transporting metal ions, it remains to be confirmed whether PtaT can indeed bind *Fn*-targeting tsRNAs.

To investigate the interaction between PtaT and tsRNAs, we ectopically expressed FLAG (DYKDDDDK) tagged PtaT (FLAG-PtaT) in *Escherichia* *coli*. In agreement with the RNA affinity pull-down assay from the total lysate of *Fn* strains, 5′ or 3′-biotinylated tsRNA-000794 and tsRNA-020498 effectively pulled down FLAG-PtaT from the total lysate of *E. coli* overexpressing the target protein, while the scrambled control had minimal binding. To rule out the possibility that tsRNAs may indirectly bind FLAG-PtaT in the total lysates of *E. coli*, we further purified recombinant FLAG-PtaT from *E. coli*. It was demonstrated that when immobilized on streptavidin magnetic beads, biotinylated tsRNA-000794 and tsRNA-020498 but not the scrambled control RNA directly pulled down PtaT in vitro (Fig. 1d). Conversely, we used purified FLAG-tagged PtaT along with magnetic beads conjugated with anti-FLAG antibodies as the bait to pull down tsRNA-000794, tsRNA-020498 or the scrambled control, and performed quantitative PCR to measure the levels of remaining free RNAs in the supernatant. As shown in SI Fig. S2, both tsRNAs exhibited interactions with the target proteins. Importantly, in this latter assay, the absence of biotin labeling, 2′-O-methylation and phosphorothioate bond in the two tsRNAs suggested that the direct binding between tsRNA and its target is dependent on the specific sequence rather than chemical modifications.

Having validated that PtaT can indeed interact with tsRNA-000794 and tsRNA-020498 of either chemically modified or naturally occurring ones, we next sought to examine whether PtaT may also bind to their DNA counterparts. To this end, we synthesized three 5′ biotinylated DNA oligos with identical chemical modifications corresponding to tsRNA-000794, tsRNA-020498, and the scrambled RNA, respectively. We found that the recombinant *Fn* PtaT protein purified from *E. coli* can only bind tsRNA-000794 and tsRNA-020498 but not their DNA counterparts (Fig. 1e). Meanwhile, we compared biotinylated tsRNA to DNA oligos of the same sequences by performing the same pulldown assays in *Fn* ATCC 23726 total lysate, followed by silver staining and Mass spectrometry. Consistent with the direct binding experiment using purified PtaT, Mass spectrometry results demonstrated that tsRNA-000794 but not its DNA counterpart can pull down PtaT from *Fn* ATCC 23726 (SI Fig. S3). In addition to the scrambled control RNA, we found that two additional piwi-interacting RNAs (piRNAs) commonly found in human saliva did not pull down PtaT from the *Fn* total lysate. In summary, through three different pulldown experiments, including ectopic expression of FLAG-tagged PtaT in *E. coli* and *Fn*, respectively, as well as the use of purified PtaT, we showed that tsRNA-000794 and tsRNA-020498 can interact with PtaT in a highly sequence- and RNA-specific manner.

### Knocking out *ptaT* interfered with the antimicrobial efficacy of *Fn*-targeting tsRNAs

Having demonstrated that PtaT is a possible RNA-binding protein for tsRNA-000794 and tsRNA-020498 experimentally, we next explored the roles of PtaT in tsRNA-mediated growth inhibition of *Fn*. To delete *ptaT* in *Fn* 23726, we first created a *galK* mutant strain (Δ*galK*) in *Fn* ATCC 23726 background so that we could use *galK* gene as a counter-selectable marker^28,29^ to generate marker-less mutants.^30^ After obtaining the *Fn* ATCC 23726 Δ*galK* mutant, which did not display any discernible growth defect compared to wildtype, a suicide vector carrying the cloned *Fn galK* gene and sequences flanking the *ptaT* gene was designed to enable a double crossover-mediated removal of the *ptaT* gene in the Δ*galK* strain background through *galK*-mediated counterselection with 2-deoxy-galactose (2-DG). The successful deletion of both *galK* and *ptaT* genes was verified by colony PCR and Sanger sequencing (SI Fig. S4), resulting in the *Fn* Δ*galK* Δ*ptaT* mutant.

**Fig. 2:**
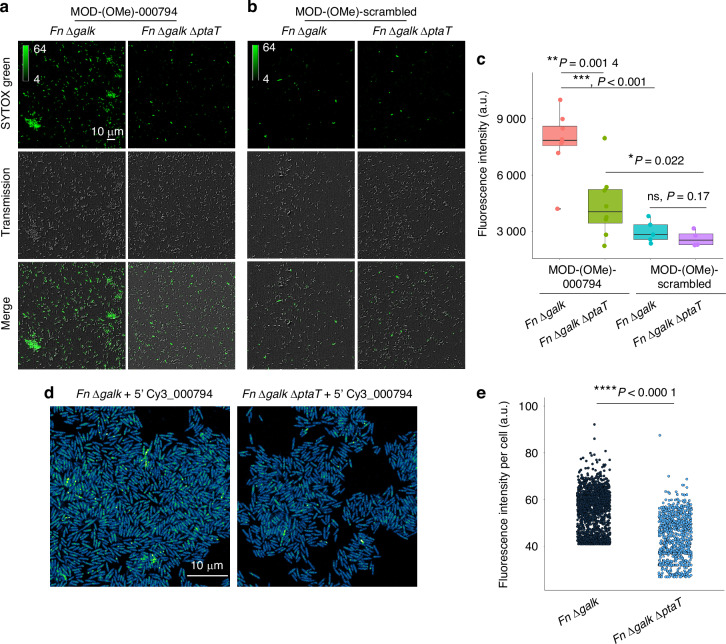
P-type ATPase transporter (PtaT) plays an important role in the internalization and antimicrobial effect of modified tsRNAs on *Fusobacterium nucleatum* ATCC 23726. **a**, **b** *Fn* Δ*galK* and *Fn* Δ*galK* Δ*ptaT* were treated with 500 nM MOD-(OMe)-000794 (**a**), and MOD-(OMe)-scrambled (**b**) for 5 h followed by SYTOX Green staining. **c** SYTOX Green quantification assay. Each dot represents the normalized raw and integrated fluorescence intensity (calculated from the fluorescence channel) to the total area of bacteria (calculated from the transmission channel), given a certain field of view (FOV). Multiple dots within a group were the FOVs chosen randomly from three biological replicates. Statistical analysis was achieved by Student’s unpaired *t*-test. ns, not significant, *P < 0.05, **P < 0.01, ***P < 0.001. **d** Visualization of internalized 5′ Cy3_000794 by *Fn* Δ*galK* and *Fn* Δ*galK* Δ*ptaT* through super-resolution Airyscan confocal microscopy. *Fn* Δ*galK* and *Fn* Δ*galK* Δ*ptaT* were incubated with 5′ Cy3_000794 for 5 h, followed by imaging. **e** Scatter plot showing the single-cell intracellular fluorescence intensity from 5′ Cy3_000794 between *Fn* Δ*galK* and *Fn* Δ*galK* Δ*ptaT*. ****P < 0.000 1

We first observed that when cultured in a standard rich medium (Columbia Broth medium), although not statistically significant, *Fn* Δ*ptaT* consistently displayed lower OD when reaching the late log/early stationary phase compared to the parent strain (SI Fig. S5). We then treated *Fn* Δ*galK* and *Fn* Δ*galK* Δ*ptaT* with chemically modified tsRNA-000794 (MOD-(OMe)-000794) and the scrambled control (MOD-(OMe)-scrambled), followed by examining the antimicrobial efficacy of tsRNAs on *Fn* through a SYTOX Green assay described previously.^26,31^ MOD-(OMe)-000794 was used as it displayed enhanced potency while maintaining specificity.^26^ As expected, MOD-(OMe)-000794 induced significant cell death in *Fn* Δ*galK*, indicated by a large portion of cells exhibiting green fluorescence (Fig. 2a). In comparison, *Fn* Δ*galK* Δ*ptaT* was relatively resistant to tsRNA-mediated growth inhibition compared to *Fn* Δ*galK* (Fig. 2a). MOD-(OMe)-scrambled control did not exert an inhibitory effect towards both *Fn* Δ*galK* and *Fn* Δ*galK* Δ*ptaT* (Fig. 2b), as evidenced by very few SYTOX Green-positive bacteria. Quantification of integrated SYTOX Green fluorescence signal after normalization of the total bacterial area further underscored that PtaT indeed plays an important role in mediating tsRNA-induced growth inhibition of *Fn* (Fig. 2c).

To query whether the increased resistance to tsRNA in *Fn* Δ*galK* Δ*ptaT* is due to the reduced tsRNA intake, we fluorescently labeled the tsRNAs with Cy3 at the 5′ end and then treated both *Fn* Δ*galK* and *Fn* Δ*galK* Δ*ptaT* with Cy3-tagged tsRNA-000794. Super-resolution Airyscanning fluorescence microscopy was then applied to examine the intracellular accumulation of tsRNA. As shown in Fig. 2d, Cy3-tagged tsRNA-000794 was taken up by *Fn* Δ*galK*, resulting in punctate fluorescence signals intracellularly, as evidenced by ‘bright dots’ (loci) indicative of subcellular accumulation of tsRNA. In contrast, by knocking out *ptaT*, the intracellular accumulation of Cy3-tagged tsRNA-000794 was significantly reduced (*P* < 0.000 1). This difference was quantified by comparing the single-cell fluorescence intensities of intracellular Cy3-tagged tsRNA-000794 between *Fn* Δ*galK* and Δ*galK* Δ*ptaT* (Fig. 2e). In summary, these data provide genetic and phenotypic evidence on the roles of PtaT in tsRNA uptake and growth inhibition in *Fn*. It is worth noting that the deletion of ptaT did not completely abolish the intake of tsRNA, suggesting PtaT is not the sole genetic determinant involved in tsRNA uptake.

### Knocking out *ptaT* altered the global RNA profiles of *Fn*

**Fig. 3:**
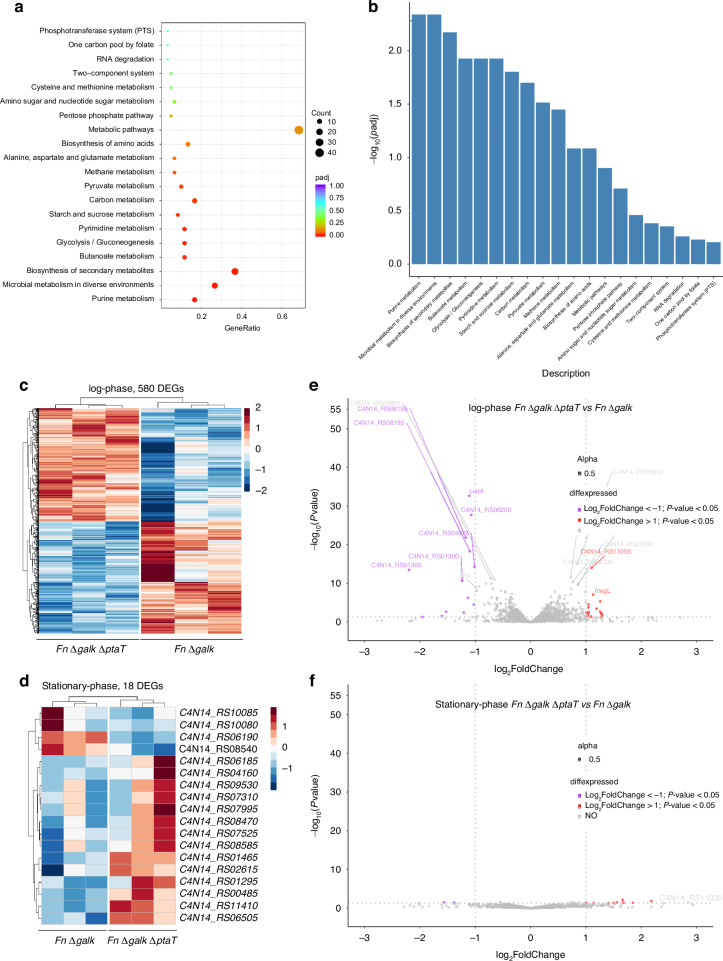
Transcriptomic analysis and comparison between *Fn* Δ*galK* and *Fn* Δ*galK* Δ*ptaT* in both log-phase and stationary-phase. **a**, **b** Clusters of orthologous groups (COG, **a**) and quantification of differentially expressed genes (**b**) from log-phase *Fn* Δ*galK* Δ*ptaT* relative to *Fn* Δ*galK*. **c**, **d** Heatmaps showing the global differentially expressed genes for log-phase (**c**) and stationary-phase (**d**) *Fn* Δ*galK* and *Fn* Δ*galK* Δ*ptaT*. Each heatmap includes triplicate RNA-seq samples for the indicated *Fn* Δ*galK* and *Fn* Δ*galK* Δ*ptaT*. The coloring indicates Log_2_FoldChange of the selected samples, while red and blue denote up- and down-regulation, respectively. The DESeq2 method (*P*-value ≦ 0.05) was applied to generate the heatmap. **e**, **f** Volcano plots showing the transcriptomic changes of log-phase (**e**) and stationary-phase (**f**) *Fn ΔgalK ΔptaT* relative to *Fn* Δ*galK*. Red and magenta dots indicate significantly upregulated and downregulated genes, respectively, and grey dots indicate genes with no significant changes. Significantly differentially regulated genes are characterized by an absolute fold change >2 (downregulated log2 < −1, upregulated log2 > 1; vertical dashed line) and *p*-value < 0.05 (horizontal gray dashed line)

Our data so far demonstrate the role of PtaT in mediating the inhibitory effect of host-derived tsRNAs against *Fn*. To further investigate the additional biological role of PtaT in *Fn*, we performed RNA-seq to identify the differentially expressed genes (DEGs) in *Fn* Δ*galK* Δ*ptaT* compared to *Fn* Δ*galK* in both log and early stationary phases. Gene expression levels were compared between *Fn* Δ*galK* and *Fn* Δ*galK* Δ*ptaT* during log-phase growth in three biological replicates. A total of 580 DEGs with a false discovery rate (FDR)-adjusted *p*-value < 0.05 are identified and presented by heatmaps and a volcano plot (Fig. 3). KEGG enrichment scatter plot of DEGs (Fig. 3a) and quantification of differentially expressed genes analysis (Fig. 3b) showed that purine metabolism represents one of the most significantly downregulated pathways in *Fn* Δ*galK* Δ*ptaT* (Fig. 3a, b). Other significantly downregulated pathways include the biosynthesis of secondary metabolites; pyrimidine metabolism, pyruvate metabolism; alanine, aspartate and glutamate metabolism (Fig. 3e). Log-phase *Fn* Δ*galK* Δ*ptaT* also displayed increased expression of glycerophosphoryl diester phosphodiesterase and genes related to methionine metabolism (Fig. 3e).

On the contrary, only 18 DEGs were identified between *Fn* Δ*galK* and *Fn* Δ*galK* Δ*ptaT* during stationary-phase growth (Fig. 3d). As shown in the volcano plot (Fig. 3f) and the clusters of orthologous group plot (SI Fig. S6), two DEGs displayed reduced expression in *Fn* Δ*galK* Δ*ptaT* compared to *Fn* Δ*galK*: C4N14_RS10085 (related to glutamine metabolism); C4N14_RS10080 (related to carbamoyl-phosphate metabolism). Nine DEGs showing upregulated gene expression are related to lipopolysaccharide biosynthesis (C4N14_RS09530), tRNA activity (C4N14_RS01465), and histidine phosphatase (C4N14_RS08585). The drastic difference in gene expression pattern between *Fn* Δ*galK* Δ*ptaT* and *Fn* Δ*galK* in log-phase and stationary-phase bacteria agreed with previous studies: the metabolism-linked genes are highly expressed when cells are actively growing and get turned off or downregulated when the cells enter the stationary phase.^32–34^ Taken together, the transcriptomic data highlight the important role that PtaT plays in shaping the global metabolic profiles of *Fn*, particularly during its actively growing state. The data are in agreement with the finding that *Fn ΔptaT* mutants display reduced growth, albeit not statistically significant, in the late log phase (SI Fig. S5).

### RNA quantification and Raman spectroscopy revealed the PtaT-dependent reduction of nucleic acid levels in *Fn*

Purine and pyrimidine are involved with the major energy carriers, and they are the subunits of nucleic acids.^35,36^ Since our transcriptomic data indicates potentially impaired purine synthesis in *Fn* Δ*galK* Δ*ptaT* in the log phase, we wondered whether the reduced gene expression of purine synthesis-related genes in the log phase would later reduce the abundance of nucleic acids inside bacteria. To answer this question, we first analyzed and compared bulk RNA levels in the stationary-phase cells. It was found that the level of bulk RNA extracted from equal numbers of *Fn* Δ*galK* was ~two-fold higher than that of *Fn* Δ*galK* Δ*ptaT* (*P* = 0.037, SI Fig. S7). Interestingly, there was no significant difference between log-phase *Fn* Δ*galK* Δ*ptaT* and *Fn* Δ*galK* in the total RNA levels (*P* = 0.77). These findings suggested a delayed response at the RNA levels following decreased gene expression related to purine/nucleic acid metabolism.

**Fig. 4:**
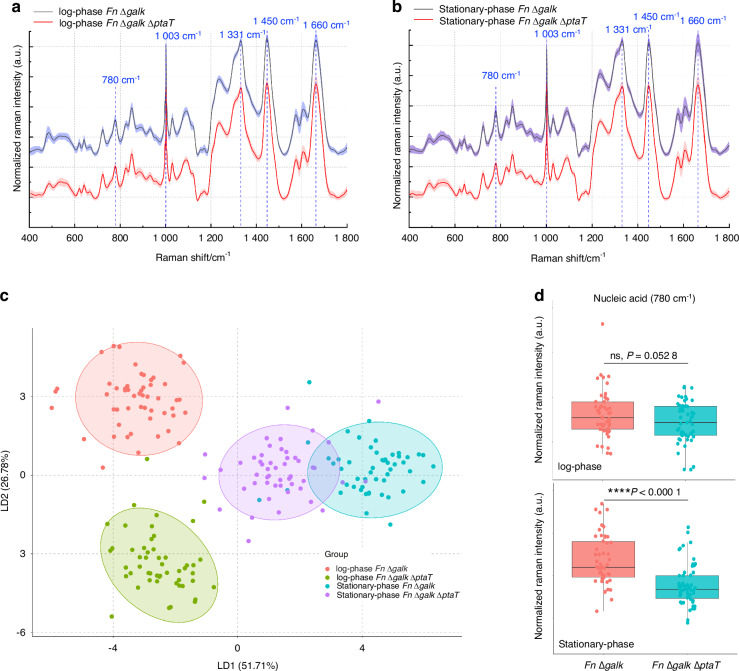
Spectroscopic characterization of both log-phase and stationary-phase *Fn* Δ*galK* and *Fn* Δ*galK* Δ*ptaT* by label-free Raman spectroscopy. **a**, **b** Averaged Raman spectra of log-phase (**a**) and stationary-phase (**b**) *Fn* Δ*galK* and *Fn* Δ*galK* Δ*ptaT*. Each scenario was averaged from 50 spectra over three biological replicates. The shaded area represents the standard deviation from the measurements. Peaks of interest were highlighted by dashed blue lines and designated with corresponding Raman shifts. **c** Linear discrimination analysis (LDA) of 200 Raman spectra from log-phase and stationary-phase *Fn* Δ*galK* and *Fn* Δ*galK* Δ*ptaT*. The plot was shown through the display of LD2 versus LD1. 95% confidence intervals were outlined by colored ellipses. **d** Quantification of the amount of nucleic acids from both log-phase and stationary-phase *Fn* Δ*galK* and *Fn* Δ*galK* Δ*ptaT*. Quantification of nucleic acids was calculated through the integration of Raman intensity from 770 to 789 cm^−1^. Statistical analysis was achieved by the Student’s unpaired *t*-test: ****P < 0.000 1, ns not significant

To further corroborate the difference in total bulk RNA extraction experiments, we performed Raman spectroscopy to understand how PtaT-depletion impacted *Fn* Δ*galK* at the molecular and cellular levels. Since Raman spectroscopy has been widely used to provide insights into the chemical makeup of biological samples at the single-cell level,^37–39^ we sought to obtain spectroscopic vibrational information of intracellular biomolecules from both the log- and stationary-phase *Fn* Δ*galK* and *Fn* Δ*galK* Δ*ptaT*. As shown in Fig. 4a, b, both log- and stationary-phase *Fn* Δ*galK* and *Fn* Δ*galK* Δ*ptaT* exhibited typical Raman peaks: 720/780 cm^−1^ (DNA/RNA); 1 003 cm^−1^ (phenylalanine); 1 240 cm^−1^/1 450 cm^−1^/1 660 cm^−1^ (Amide III/II/I peaks).

To globally map out the difference among the four groups, a multivariate data analysis approach, linear discrimination analysis^40,41^ (LDA) was utilized to model the difference among the four groups through dimensionality reduction of high-dimensional Raman spectra. Each group indeed exhibited a distinct cluster in the LDA plots (Fig. 4c and SI Fig. S8). Raman band at 780 cm^−1^ is related to nucleic acids due to the cytosine/uracil ring breathing.^42^ We then quantified the amount of nucleic acids based on the integrated Raman intensity around 780 cm^−1^ given that Raman intensity is linearly proportional to the amount of biomolecules inside the samples.^43^ We found that *Fn* Δ*galK* Δ*ptaT* had significantly lower nucleic acid levels (*P* < 0.000 1) than that of the isogenic control *Fn ∆galK* in the stationary phase compared to that of the log phase (ns, *P* = 0.052 8), suggesting that the reduced purine synthesis likely limited nucleic acids level in the stationary phase (Fig. 4d). Consistent with the transcriptomic data and RNA quantification from the bulk sample (SI Fig. S7), label-free Raman spectroscopy provides another line of evidence that PtaT may affect the purine synthesis process which negatively impacts intracellular nucleic acid levels at the stationary phase.

### Knocking out *ptaT* in a *Fn* clinical tumor isolate interfered with the intake and antimicrobial efficacy of *Fn*-targeting tsRNAs

After demonstrating the involvement of PtaT in tsRNA-mediated growth inhibition in the type strain *Fn* ATCC 23726, we next explored whether similar findings can be validated in a clinical *Fn* isolate. *Fn* is a significant contributor to colorectal cancer (CRC).^44^ Since we have shown the excellent antimicrobial efficacy of MOD-(OMe)-000794 against multiple *Fn* clinical tumor isolates (CTIs),^26^ we wondered whether PtaT plays similar roles in tsRNA intake and its induced growth inhibition as observed in *Fn* ATCC 23726.

**Fig. 5:**
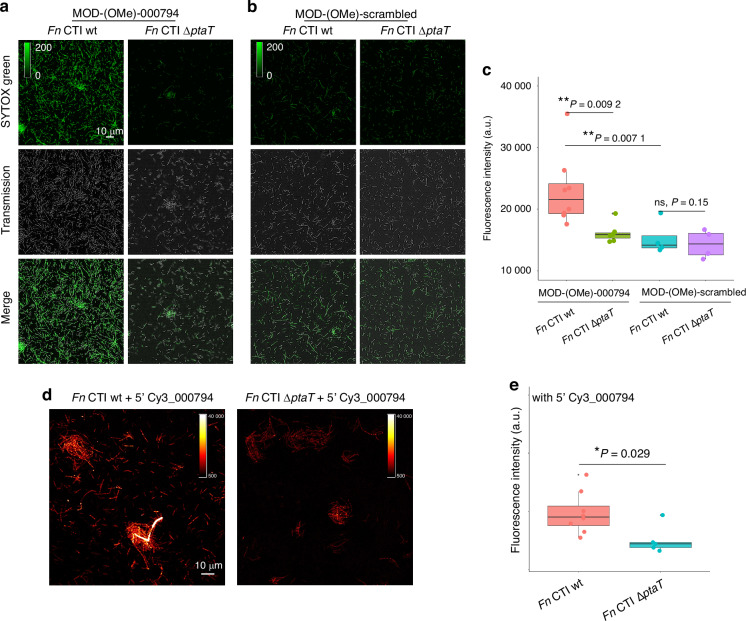
P-type ATPase transporter (PtaT) plays an essential role in the internalization and antimicrobial effect of modified tsRNAs on a *Fusobacterium nucleatum* clinical tumor isolate (*Fn* CTI-2). **a**, **b** *Fn* CTI wt and *Fn* CTI Δ*ptaT* were treated with 500 nmol/L MOD-(OMe)-000794 (**a**), and MOD-(OMe)-scrambled (**b**) for 5 h, followed by SYTOX Green staining. **c** SYTOX Green quantification was carried out by normalizing raw integrated fluorescence intensity to the areas of randomly picked bacteria, which takes into consideration both SYTOX Green-positive and -negative ones in the field of view. Statistical analysis was achieved by the unpaired Student’s *t*-test: **P < 0.01, *P < 0.05, ns not significant. **d** Visualization of internalization of 5′ Cy3_000794 by *Fn* CTI wt and *Fn* CTI Δ*ptaT* through confocal fluorescence imaging. *Fn* CTI wt and *Fn* CTI Δ*ptaT* were incubated with 5′ Cy3_000794 for 5 h, followed by confocal fluorescence imaging. **e** Quantification of fluorescence intensity by normalizing raw integrated fluorescence intensity to the areas of bacteria, as shown in (**d**). Statistical analysis was achieved by the Student’s unpaired *t*-test: *P < 0.05

To test this, we chose *Fn* clinical tumor isolate (CTI)-2 because CTI-2 expresses a membrane protein, Fap2, that mediates the adhesion of *Fn* to colon cancer cells overexpressing Gal-GalNAc to promote the development of CRC.^45^ Additionally, we also found that the protein sequences for PtaT are identical between CTI-2 and *Fn* ATCC 23726. For these reasons, we generated a PtaT-depleted *Fn* CTI-2 strain (*Fn* CTI Δ*ptaT*) as described in “Materials and methods”.^46^ After validating the successful knockout of *ptaT*, we examined the inhibition efficacy of tsRNAs on *Fn* CTI-2 wildtype (*Fn* CTI wt) and *Fn* CTI Δ*ptaT* through the SYTOX Green assay. MOD-(OMe)-000794 induced apparent cell death in the case of *Fn* CTI wt, indicated by a large portion of cells exhibiting green fluorescence (Fig. 5a) and consistent with our previous study.^26^ Similar to findings in *Fn* 23726 Δ*galK* Δ*ptaT*, we also observed that *Fn* CTI Δ*ptaT* was relatively resistant to tsRNA-mediated growth inhibition compared to *Fn* CTI wt (Fig. 5a). MOD-(OMe)-scrambled didn’t exert an inhibitory effect towards both *Fn* CTI wt and *Fn* CTI Δ*ptaT* (Fig. 5b), as evidenced by few SYTOX Green-positive bacteria. Quantification of integrated SYTOX Green fluorescence signal after normalization of the total bacterial area further underscored that PtaT indeed plays an indispensable role in mediating tsRNA-mediated growth inhibition of a *Fn* clinical tumor isolate (Fig. 5c).

Furthermore, the deletion of *ptaT* in CTI seemed to affect tsRNA intake. As shown in Fig. 5d, Cy3-tagged tsRNA-000794 was densely accumulated intracellularly, as indicated by the formation of multiple loci with strong signal intensity. By knocking out *ptaT*, the intracellular accumulation of Cy3-tagged tsRNA-000794 was significantly reduced in the *Fn* CTI Δ*ptaT* compared to *Fn* CTI wt (*P* = 0.029, Fig. 5e). This difference was documented by the comparison of the normalized Cy3 fluorescence intensity between *Fn* CTI wt and *Fn* CTI Δ*ptaT*. These data provide further evidence showing the important role of PtaT in tsRNA-mediated growth inhibition against a wide range of *Fn* strains, including clinically relevant ones.

### AlphaFold 3 simulates the interaction between tsRNA-000794 and PtaT

**Fig. 6:**
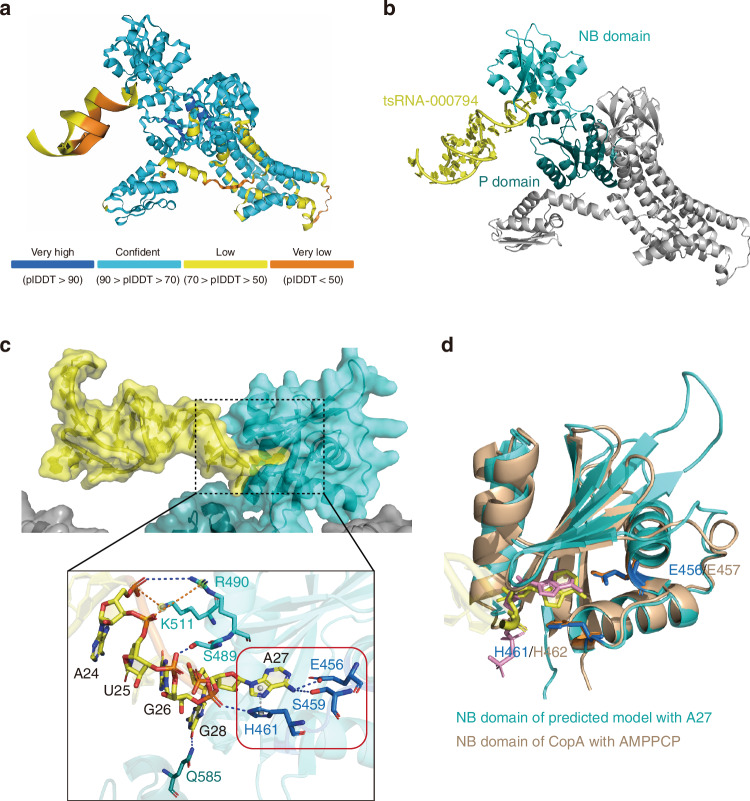
Structural analysis of the predicted complex model of tsRNA-000794 and PtaT. **a** The predicted complex model of tsRNA-000794 and PtaT by AlphaFold 3. The color of the model presents the confidence of the prediction, as shown in the bar below. **b** Cartoon view of the predicted complex model. The tsRNA-000794 is shown in yellow, and the PtaT protein is shown in gray, with its Nucleotide Binding (NB) domain in cyan and Phosphorylation (P) domain in deep teal. **c** Structural analysis of the binding pocket of the predicted model. Upper, binding region of tsRNA-000794 and PtaT. Both RNA and protein are shown in a cartoon with a surface view. Colors are the same as those in (**b**). Lower, detailed Interactions between tsRNA and NB domain/P domain of PtaT protein. The core pocket is highlighted in red brick. Nucleotide acids are in yellow. Key residues are shown in sticks. Residues in core pocket of NB domain are in blue, in extended region in NB domain are in cyan, and in P domain is in deep teal. Dotted lines represent interactions. Among them, gray indicates π–π interaction, blue indicates hydrogen bonding, and orange indicates salt bridge. **d** The superimposition of the predicted model (cyan) and CopA (wheat, PDB code: 3A1C). Only the NB domain is shown here for both proteins. A27 in tsRNA-000794 binds to the core pocket of PtaT, which is in yellow stick, and AMPPCP, an ATP analog that binds to CopA, is in pink stick. Key residues in the core pocket are also shown in stick, they are E456 and H461 of PtaT in blue, and E457 and H462 of CopA in orange. RMSD of NB domain between PtaT and CopA is 1.527 Å

Our biochemical and genetic data (Fig. 1, Fig. 2, Fig. 5) suggest that PtaT may act as an RNA-binding protein involved in the intake of *Fn*-targeting tsRNAs. However, none of the PtaT characterized thus far has been shown to be involved in sRNA transport. To obtain additional information on the potential interaction between *Fn*-targeting tsRNA and PtaT, we employed AlphaFold 3 to predict biomolecular interaction between PtaT from *Fn* ATCC 23726 and tsRNA-000794.^47^ We downloaded the full protein sequence of PtaT for *Fn* ATCC 23726 from the UniProt website that corresponds to the protein identified by mass spectrometry for the tsRNA pulldown assay and chose tsRNA-000794 due to its higher uptake by *Fn* compared with tsRNA020498.^26^ We then utilized AlphaFold 3 to predict the structure model of the tsRNA-000794—PtaT protein complex (Fig. 6a). The prediction results suggest the presence of RNA-binding domains with high confidence. Of note, P-type ATPase has a conserved Nucleotide Binding domain (NB domain), which is in the cytoplasm and is responsible for ATP binding and hydrolysis.^48^ It also possesses a characteristic Phosphorylation domain (P domain), which is closely related to the NB domain and involved in the phosphorylation reaction following ATP hydrolysis.^49^ Consistent with the above biochemical data, AlphaFold3 prediction suggested tsRNA-000794 may interact with *Fn* PtaT by contacting both the NB domain and the P domain (Fig. 6b). A detailed analysis of the predicted PtaT-tsRNA interaction reveals a core pocket and an extended region in PtaT for tsRNA binding (Fig. 6c).

The binding mode of the tsRNA-000794 to PtaT resembles the classical binding mode of ATP to P-type ATPases. For example, CopA proteins, a class of P-type ATPases that translocate Cu^2+^, have had their crystal structures solved, including those containing the ATP analogue—AMPPCP.^50^ The CopA–AMPPCP complex structure (PDB code: 3A1C) can be used as a model to study ATP binding to P-type ATPases, serving as the homologue to the PtaT protein. The superimposition of the NB domains of the PtaT protein and CopA reveals a high degree of similarity, especially at the core pocket site. Correspondingly, tsRNA-000794 exhibits a similar mode of insertion into the core pocket as AMPPCP (Fig. 6d).

We further predicted the interactions between *Fn* PtaT and tsRNA scrambled control, or a DNA oligo with sequences identical to tsRNA-00079, referred to as tsDNA-000794. As shown in SI Fig. S9a, while the scrambled control adopted a relatively stable conformation within the complex, it lacks the specificity to bind to the pocket in the NB domain, as seen with the tsRNA-000794. We further simulated the interaction between tsDNA-000794 and PtaT, and we found no binding between them (SI Fig. S9b).

To verify whether amino acids depicted in Fig. 6c are crucial for the interaction with tsRNAs, we performed alanine scanning followed by an Electrophoretic Mobility Shift Assay (EMSA). In the AlphaFold 3-predicted binding model, key residues E456, S459, H461, and K511 play crucial roles: E456, S459, and H461 form hydrogen bonds and/or π–π interactions within the binding pocket, while K511 interacts with RNA phosphate groups in the extended region (Fig. 6). Alanine substitution of E456 and H461 rendered the protein insoluble, likely due to their role in structural stability. To preserve protein folding, we introduced milder mutations (E456L and H461F) to retain some side-chain properties. We tested single mutants (E456L, S459A, H461F, and K511A), as well as double and multi-point mutants (E456L/H461F, E456L/K511A, E456L/S459A/H461F, and E456L/S459A/H461F/K511A). All mutated proteins were validated by fast protein liquid chromatography (FPLC) for structural integrity and tested in EMSA with annealed tsRNA-000794.

For the EMSA experiment, samples were stratified into three groups (SI Fig. S10). The first group was designed to validate the RNA-binding capacity of the wild-type NB domain (NB WT). To improve solubility, both wild-type and mutant NB variants were N-terminally fused to **G**lutathione **S**-**t**ransferase (GST), a widely used solubility-enhancing tag. To eliminate potential artifacts from the tag, a control assay confirmed that GST alone does not bind tsRNA (SI Fig. S10a, left). With tsRNA held constant at 2 μmol/L, a distinct mobility shift was observed upon incubation with GST-NB WT, at ~6.25 μmol/L protein concentration, indicating a strong interaction (SI Fig. S10a, right). The second group included single-point NB domain mutants (SI Fig. S10b). Substitutions E456L and H461F, despite partially retaining side-chain properties, markedly reduced tsRNA binding, with shift bands appearing only at ~25 and ~50 μmol/L protein concentration lanes, respectively. The S459A variant exhibited similarly reduced affinity, whereas the K511A mutation completely abolished binding. The third group comprised double and multi-site mutants (SI Fig. S10c), none of which demonstrated detectable tsRNA interaction under the tested conditions. Collectively, these data demonstrate that the wild-type NB domain binds to tsRNA directly and specifically, while certain point mutations severely compromise or eliminate this interaction. These findings are consistent with the predicted interface of AlphaFold 3 modeling.

In summary, AlphaFold 3 prediction provides further evidence supporting the specific binding between PtaT and tsRNA compared to the scrambled control and the DNA counterpart, which agrees with our biochemical and genetic data (Fig. 1, Fig. 2, Fig. 5).

## Discussion

Small regulatory noncoding RNAs (sRNAs), including transfer RNA-derived sRNAs (tsRNAs), are a class of regulatory elements that have been identified in prokaryotes^51^ and eukaryotes^52^ and implicated in gene regulation. Recent studies further revealed the potential role of sRNAs in interspecies and cross-domain interactions^53^ with increasing lines of evidence showing that host-derived sRNAs such as fecal miRNAs can modulate the compositions of host-associated microbiota.^54,55^ We recently reported tsRNA-mediated cross-kingdom interplay in the context of human oral microbiome-host interactions. We demonstrated that when challenged with *Fn*, the oral epithelial cells release *Fn*-targeting tsRNAs that selectively inhibit the growth of *Fn* via their ribosome-targeting function.^26^ However, the bacterial genetic determinants involved in tsRNA-mediated growth inhibition remain to be fully characterized. In this work, through biochemical analysis, we identified a fusobacterial protein, annotated as a putative membrane-associated P-type ATPase transporter (named PtaT), that is capable of binding tsRNAs in a sequence-dependent manner (Fig. 1, SI Fig. S10). More importantly, our genetic and phenotypic data strongly support PtaT’s role in mediating the growth inhibition of *Fn*-targeting tsRNAs (Figs. 2–5).

One intriguing question is whether PtaT may serve as a transporter for the uptake of extracellular host-derived tsRNAs. Over the last decades, transmembrane RNA importer proteins in eukaryotes, such as Systemic RNA Interference Deficiency-1 (SID-1)^56^ in *Caenorhabditis elegans*, and its homologs SIDT1^57^ and SIDT2^58^ in mammals, have been identified that facilitate internalization of extracellular sRNAs for intercellular or cross-kingdom gene modulation. However, little progress has been made toward elucidating how host-derived sRNAs may enter bacteria in a cross-kingdom fashion. P-type ATPases are a large group of evolutionarily related integral membrane proteins found in bacteria, archaea and eukaryotes. Most of the P-type ATPases characterized so far are active pumps that couple ATP hydrolysis to the transport of diverse substrates, ranging from H^+^, metal cations, phospholipids, to polyamines.^59,60^ Our RNA affinity pulldown and Cy3-tsRNA uptake experiments in *ptaT* mutants (Fig. 1, Fig. 2, Fig. 5) suggest that PtaT may play a key role in the binding and uptake of *Fn*-targeting tsRNAs. Additionally, the alleviated tsRNA-induced growth inhibition in *ptaT* mutant could be due to the reduced tsRNA uptake, further supporting PtaT is involved in transporting tsRNA.

While PtaT can bind tsRNAs experimentally (Fig. 1), AlphaFold3 prediction and alanine scanning (Fig. 6, SI Fig. S10) indicated that the binding likely happens at the cytoplasmic nucleotide-binding (N) domain. Furthermore, the predicted high-affinity binding of *Fn*-targeting tsRNA to the ATP binding site in PtaT also seems to argue against PtaT being a transporter for tsRNA intake, as the occupation of the ATP binding site by tsRNA will likely abolish PtaT’s function, which is predicted to be driven by hydrolysis of ATP. Thus, while our data strongly indicate PtaT is a tsRNA-binding protein and involved in tsRNA-mediated growth inhibition, it remains to be determined if it is a bona fide sRNA transporter. More direct evidence for the role of PtaT in tsRNA intake needs to come from detailed structural characterization of PtaT–tsRNA complex using X-ray crystallography or cryo-electron microscopy, as well as directly testing the ability of PtaT for cross-membrane tsRNA transport using an in vitro liposome membrane system.^61^

If PtaT is not a bona fide transporter for tsRNA, the observed reduced tsRNA intake in PtaT-deletion mutant (Fig. 2, Fig. 5) could be an indirect effect via an unknown pathway, and the binding of tsRNA to PtaT may trigger downstream yet-to-be-determined functions to impact bacterial growth. This intriguing question warrants further investigation.

Compared to wildtype, *Fn* Δ*ptaT* mutant displayed a significantly downregulated expression in purine metabolism-related genes during its log-phase growth, followed by a drastic reduction in intracellular nucleic acid levels at the stationary phase (Fig. 4). The data agree with the finding that the *Fn* Δ*ptaT* mutant displays reduced growth, albeit not statistically significant, in the late log phase (SI Fig. S5). Interestingly, our previous study^26^ also revealed that treatment of *Fn* with *Fn*-targeting tsRNA markedly downregulated the same purine metabolism pathway compared to the scrambled control. While further investigation is warranted, it is tempting to speculate that PtaT may contribute to purine biosynthesis, especially during the stationary phase. This function could be negatively impacted as a result of the competitive binding of PtaT by *Fn*-targeting tsRNAs, making PtaT one of the cellular targets of tsRNAs and contributing to tsRNA-induced growth inhibition.

Given the abundance of extracellular host-derived sRNAs and their suspected role in microbial-host interaction, understanding host-derived tsRNA uptake mechanisms and the mode of action in *Fn* pathogenesis may have broader implications for developing targeted therapeutics and interventions against *Fn*-associated diseases. Future studies are warranted to determine if PtaT is a bona fide transporter for tsRNA, and it may also present a potential target mediating tsRNA-induced growth modulation. Nevertheless, by identifying PtaT as a tsRNA-binding protein and demonstrating its involvement in tsRNA-mediated growth modulation, our study offers new insights into sRNA-mediated host-pathogen interplay in the context of oral microbiome-host interactions.

## Materials and methods

### Chemicals

All chemicals and cell culture broth were purchased from Fisher Scientific International Inc. (Cambridge, MA, USA) unless otherwise noted, and were of the highest purity or analytical grade commercially available. DNA and RNA oligos were ordered from Sigma Aldrich (St. Louis, MO, USA) and Integrated DNA Technologies (Coralville, IA, USA). All molecular cloning reagents, including restriction enzymes, competent cells, and the Gibson assembly kit, were purchased from New England Biolabs (Ipswich, MA, USA).

### Bacterial strains (summarized in Supplementary Table S2) and growth conditions

*Fusobacterium nucleatum* ATCC 23726, 25586, 10953, *Streptococcus mitis* ATCC 6249, and *Porphyromonas gingivalis* ATCC 33277 were purchased from the American Type Culture Collection (Manassas, VA, USA). *F. nucleatum* colon tumor isolate was a general gift of Dr. Wendy Garrett at the Harvard T.H. Chan School of Public Health. *F. nucleatum* strains and *P. gingivalis* were cultured in liquid Columbia broth (CB) or on CB agar plates containing 5% defibrinated sheep blood, and incubated at 37 °C in an anaerobic chamber (Sheldon Manufacturing, Cornelius, OR, USA) containing 5% H_2_, 10% CO_2_, 85% N_2_. *S. mitis* was cultured in Brain-Heart Infusion (BHI) broth. *E. coli* was cultured in lysogeny broth (LB) media and incubated at 37 °C under aerobic conditions. Thiamphenicol at 5 μg/mL (Fisher Scientific) was used for the selection and maintenance of *Fn* strains possessing pHS31. *F. nucleatum* CTI-2 strain and its derivatives were cultured in a TSPC medium comprising 3% tryptic soy broth (BD), 1% Bacto peptone, and 0.05% cysteine. Fusobacterial transformants with pBCG02-based deletion plasmid were grown overnight in tryptic soy broth supplemented with 1% Bacto peptone plus 0.25% freshly made cysteine (TSPC) broth with 5 µg/mL thiamphenicol. *E. coli* strains were grown in Luria–Bertani (LB) broth with aeration at 37 °C. *E. coli* strains carrying plasmids were grown in LB broth containing 20 µg/mL chloramphenicol.

### Plasmid construction for genetic knockout of *F. nucleatum* ATCC 23726

A list of primers is provided in Supplementary Table S3. For expression and purification of recombinant PtaT in *E. coli*, the full-length cDNA was amplified from the genomic DNA of *F. nucleatum* ATCC 23726 genome via primers B267/B268 and ligated to pSH200 via *BamH I* and *Not I* by the Gibson Assembly kit. His-tagged proteins were expressed in Rosetta (DE3) *E. coli*. For constructing a suicide vector for insertional mutagenesis of PtaT in *Fn*, a 1kb central fragment was cloned into pHS31 after *SnaBI* digestion through the Gibson assembly. For overexpression in *Fn*, a shuttle vector, pHS58, was first digested with *XhoI* and *HindIII*. The FN1529 promoter amplified from *Fn 25586*, and the full-length *ptaT* were assembled with the digested pHS58. All constructs were first cloned into NEB 5-alpha chemically competent *E. coli* and then verified by Sanger sequencing before transforming into the desired strains.

### Generation of the deletion mutant in *F. nucleatum* ATCC 23726 via electroporation

The Δ*patT* strain was generated from the following steps. Electrocompetent *F. nucleatum* ATCC 23726 Δ*galK* was prepared by growing a 10 mL culture to log phase (OD_600_ = ~0.8) followed by centrifugation at 12 000×*g* for 10 min, removal of the supernatant, and three successive washes with 750 μL of ice-cold electroporation buffer (10% glycerol, 1 mmol/L MgCl_2_ in deionized H_2_O). For each electroporation, cells were resuspended in ice-cold electroporation buffer at an OD_600_ of 12. Bacteria were transferred to ice-cold 1-mm electroporation cuvettes (Fisher Scientific), and anaerobically incubated with 1 μg (concentration >500 ng/μL) of plasmid on ice for 10 min before electroporating at a setting of 2.5 kV (25 kV/cm), 25 μF and 200 Ω using a Gene Pulser Xcell Electroporation System (Bio-Rad). Immediately after electroporation, bacteria were transferred to 1 mL of anaerobically pre-reduced CB supplemented with 1 mmol/L MgCl_2_ and then incubated anaerobically at 37 °C overnight. After outgrowth, bacteria were spun down at 12 000×*g* for 5 min, the medium was removed, and cells were spread on CB blood agar plates containing 5 μg/mL thiamphenicol. Plates were incubated in an anaerobic chamber at 37 °C for 3–7 days for colony growth to select for the first crossover step. Successful single crossover clones were verified by gDNA extraction, colony PCR and Sanger sequencing.

To enable the second step of crossover, sequencing-verified clones were inoculated in 2 mL fresh CB broth containing 0.25% 2-deoxy-d-galactose (2-DG) and incubated under anaerobic conditions overnight at 37 °C. Putative *Fn* Δ*patT* clones were selected by spreading 100 μL of diluted culture on a blood agar plate containing 0.25% 2-deoxy-d-galactose (2-DG). Plates were incubated under anaerobic conditions for 3–4 days at 37 °C. 1–3 colonies were then re-streaked on the 0.25% 2-DG agar plates for another 3 days of incubation. 8–10 single colonies were randomly picked and streaked onto blood agar plates supplemented with 0.25% 2-DG. The same colonies were simultaneously streaked onto blood agar plates containing 5 μg/mL thiamphenicol to confirm thiamphenicol sensitivity, as the loss of thiamphenicol resistance indicates the removal of the plasmid backbone. Subsequently, thiamphenicol-sensitive colonies were inoculated into 1% 2-DG CB broth and passaged 4 times in 1% 2-DG CB broth, followed by spreading onto 0.25% 2-DG blood agar plates. 10–20 single colonies were picked, expanded for genomic DNA extraction and verified by colony PCR and Sanger sequencing to confirm the double crossover knockout for *patT*.

### Plasmid construction for genetic knockout of *F. nucleatum* CTI-2

PCR primers used in this study are listed in Supplementary Table S3. For the deletion of CTI-2 gene *ptaT*, the plasmid pBCG02-CTI2-1787updn was constructed via Gibson assembly cloning according to the manufacturer’s instructions with NEBuilder HiFi DNA Assembly master mix. Briefly, 1.0 kb fragments of upstream and downstream regions were amplified by PCR using primer pairs CTI2-1787up-F/CTI2-1787up-R and CTI2-1787dn-F/CTI2 1787dn-R, respectively (Table S2). The overlapping PCR was used to ligate two PCR amplicons using primer pair CTI2-1787up-F/CTI2-1787dn-R. The fused segment was mixed in 10 µL Gibson assembly master mix solution with pBCG02 vector backbone generated by inverse PCR with primer pair pCWU6-F/pCWU6-R.^62^ A 2× PrimerSTAR® Max DNA Polymerase master mix from TaKaRa (catalog No. R045A) was used for high-fidelity PCR amplification of the gene fragments and pBCG02 backbone. The resulting plasmid pBCG02-CTI2-1787updn was further confirmed by sequencing and transformed into *F. nucleatum* CTI-2 strain by electroporation.^28^

### Deletion of gene ptaT in *F. nucleatum* CTI-2 strain

Plasmid pBCG02-CTI2-1787updn integration into the bacterial chromosome DNA by homologous recombination was selected by anaerobic growth on TSPC agar plates with 5 µg/mL thiamphenicol at 37 °C. Due to the low transformable efficiency of the CTI-2 strain, successful plasmid integration was achieved after multiple attempts. The thiamphenicol-resistant colony was next inoculated in TSPC broth without antibiotics overnight. The next day, cultures were diluted 1 000-fold, and 100 µL of aliquots were spread on TSPC agar plates supplemented with 2 mmol/L inducer theophylline. This step was designed to select cells that had lost the plasmid via the second crossover event. Ten colonies were randomly chosen and re-streaked on TSPC plates to verify thiamphenicol sensitivity and analyzed by PCR to confirm the deletion of the target gene.

### Biotinylated tsRNA affinity pulldown from bacterial lysate

Dynabeads® M-270 Streptavidin beads (ThermoFisher, Catalog# 65305) and streptavidin agarose resin (G-Biosciences, St. Louis, MO, USA) were washed according to the instructions and blocked with 1 mg/mL BSA at RT for 1 h. M-270 beads and resin were washed by 3× TBS (Tris HCl 150 mmol/L, NaCl 0.45 mol/L, pH = 7.4) and 1× TBS (Tris HCl 50 mmol/L, NaCl 0.15 mol/L, pH = 7.4) three times, respectively, and resuspended in the same volume buffer as the initial volume of beads taken from vial by 3× TBS and 1× TBS. 50 mL wild type *Fn* culture was harvested by spinning at 4 000 × rcf 15 min followed by 3 times 1× PBS washing steps. Cell pellet was rotated at RT for 20 min in 3 mL cell lysis buffer (50 mmol/L Tris HCl, 0.15 mol/L NaCl, pH 7.4, 1 mmol/L DTT, 0.5% NP-40, 50 μg/mL lysozyme and protease inhibitor cocktail EDTA free). Lysates were sonicated on ice with 5 s on and 5 s off for a total 5 min at 50% power (Soniprobe, Dawe Instruments, England) for 3–4 times until the lysate was transparent and cleared by centrifugation. Heparin was added to the clear extracts with 100 μg/mL working concentration, followed by incubation with precoated resin at 4 °C for 30 min and quantified for protein concentration by DC Protein Assay Reagent package. Before incubating with pretreated M-270 beads, 1 mmol/L MgCl_2_, 1 mmol/L ATP and 40 U/mL RNaseOUT (Thermofisher) were added to the precleared cell lysates.

5′ or 3′ Biotin-tsRNA with a final concentration of 500 nmol/L was added to 10 μL of precoated M-270 beads and incubated on ice for 30 min. After 3 times of washing with 1× TBS, the precleared lysates were aliquoted to the pretreated M-270 beads equally and incubated at RT for 2 h on the rotator. M-270 beads were washed for 10 min at RT with wash buffer (50 mmol/L Tris HCl, 0.15 mol/L NaCl, pH 7.4, 0.5% NP-40) twice, followed by 1× TBST (25 mmol/L Tris, 0.15 mol/L NaCl, pH = 7.4, 0.05% TweenTM-20) twice. 1× SDS loading buffer was added to the samples and prepared for SDS-PAGE. After SDS-PAGE, the gel was stained with the FOCUS™ FASTsilver™ kit (Bioscience, 786–240). Target bands were excised and de-stained for mass spectrometry (Thermo Q Exactive) analysis at the Barnett Institute Proteomics mass spectrometry Core Facility at Northeastern University.

### Mass spectrometry

Proteins were reduced (10 mmol/L dithiothreitol, 56 °C for 45 min) and alkylated (50 mmol/L iodoacetamide, room temperature in the dark for 1 h). Proteins were subsequently digested with trypsin (sequencing grade, Promega), at an enzyme/substrate ratio of 1:50, at room temperature overnight in 100 mmol/L ammonium acetate, pH 8.9. Trypsin activity was quenched by adding formic acid to a final concentration of 5%. Peptides were desalted using C18 SpinTips (Protea), then lyophilized and stored at −80 °C. Peptides were loaded on a pre-column and separated by reverse phase HPLC (Thermo Easy nLC1000) over a 140 min gradient before nano electrospray using a QExactive Mass Spectrometer (Thermo Fisher Scientific). The Mass Spectrometer was operated in a data-dependent mode. The parameters for the full scan MS were: resolution of 70 000 across 350–2 000 m/z, AGC 3e6, and maximum IT 50 ms. The full MS scan was followed by MS/MS for the top 10 precursor ions in each cycle with an NCE of 28 and dynamic exclusion of 30 s. Raw mass spectral data files (.raw) were searched using Proteome Discoverer (Thermofisher) and Mascot version 2.4.1 (Matrix Science). Mascot search parameters were: 10 ppm mass tolerance for precursor ions; 0.8 Da for fragment ion mass tolerance; 2 missed cleavages of trypsin; fixed modification was carbamidomethylation of cysteine; variable modification was methionine oxidation. Only peptides with a Mascot score greater than or equal to 25 and an isolation interference less than or equal to 30 were included in the data analysis. Potential interacting proteins are identified in the experimental sample after the removal of proteins in the control sample and common contaminating proteins.

### Recombinant protein production and purification

*E. coli* was cultured in 250 mL LB medium until OD_600_ reached ~0.8 followed by 1 mmol/L isopropyl-β-d-thiogalactopyranoside (IPTG) induction for 18 h at 20 °C. *E. coli* was harvested by centrifugation and resuspended in Binding Buffer (0.5 mol/L NaCl, 33 mmol/L sodium phosphate, 20 mmol/L imidazole, pH 7.6) with 1–2 mg/mL lysozyme, 1% (v/v) Triton X-100, 1 tablet of EDTA-free Protease Inhibitor Cocktail, 1 mmol/L PMSF, and Benzonase followed by gently rotation at RT for 20 min. PMSF was re-added every 30 min until the protein was bound to Nickel resin. Lysates were sonicated on ice with 5 s on and 5 s off for a total of 5 min at 50% power (Soniprobe, Dawe Instruments, England) for 1–2 times until the lysate was not viscous and cleared by centrifugation for 1 h at 12 000 × rcf, 4 °C. 1 mL Nickel resin was washed with deionized H_2_O two times followed by the binding buffer washing. After the sonicated media was fully centrifuged, the supernatant was poured into 15 mL conical tube with Nickel resin, and 1% Triton-114 was added to the supernatant. After 1 h incubation at 4 °C, the resin was washed via the binding buffer containing 1% Triton-114 three times at 4 °C, 30 min per wash. Resin was transferred to the polypropylene column (Bio-Rad) and eluted with Elution Buffer (0.5 mol/L NaCl, 33 mmol/L sodium phosphate, 250 mmol/L imidazole, pH 7.6). The protein elute was concentrated to ~ 0.5 mL by 10 kD MWCO Spin Column, and then loaded onto a size exclusion column, ENrich™ SEC 650 10 × 300 (Bio-Rad), which was connected to the NGC Medium-Pressure Liquid Chromatography System (Bio-Rad). The protein concentration was monitored with OD_280_, and a fraction collector (Bio-Rad) was used to collect samples at 0.5 mL per fraction for SDS-PAGE analyses. Only fractions containing the desired protein without any impurity were pooled for downstream binding assays. After FPLC, the protein samples were added with 1 mmol/L DTT, and stored at −80 °C before use.

### Direct binding assay for tsRNA and purified proteins

Dynabeads® M-270 Streptavidin beads were prepared according to the manual. The concentration of purified proteins was measured by Pierce™ Rapid Gold BCA Protein Assay Kit. Purified PtaT was adjusted to 1 μg per reaction by adjusting buffer (50 mmol/L Tris HCl, 0.15 mol/L NaCl, pH 7.4, 0.1% NP-40, 1 mmol/L ATP, 1 mmol/L MgCI_2_, 100 μg/mL heparin,1 mg/mL BSA) and incubated with biotinylated tsRNA pretreated beads at RT for 2 h followed by washing steps as above described. 2× SDS loading buffer was added to the washed beads for Western Blot analysis.

### Fluorescence microscopy of Cy3 tsRNA-labeled Fn strains

Cy3-labeled tsRNA was reconstituted in 1× TBS containing 0.1 mmol/L EDTA for imaging. Overnight-grown *Fn* strains were diluted to OD_600_ = 0.1 and treated with 500 nmol/L of Cy3-labeled tsRNA-000794, and scrambled RNA for 5 h in an anaerobic chamber. Labeled bacteria were then washed with 1× PBS three times at a centrifuge speed of 17 000×*g* for 10 min. Washed samples were then sandwiched between a cover glass and poly-l-lysine-coated cover slide. Samples were then immediately imaged by a ZEISS LSM 800 confocal microscope with a fast Airyscan detector (with 120 nm lateral resolution and 350 nm axial resolution). To ensure the image quality, we utilized a 63× Plan-Apochromat NA = 1.4 oil immersion objective. Samples were excited at a wavelength of 514 nmol/L with a 10% power and detected in the range of 550–600 nm. To quantify the fluorescence intensity from the same sample patch, the dynamic range was adjusted to be the same under a channel-mode confocal modality. To have a clear visualization of Cy3-tsRNA incorporation at the subcellular level, super-resolution by Airyscan was achieved at a gain of 800 V. Images were visualized and analyzed by FiJi (NIH), and quantitative analysis was conducted by R.

### Western Blotting

The samples were first separated by 10% SDS-PAGE gel and then transferred to a nitrocellulose membrane (Fisher Scientific). The membranes were incubated with the Anti-FLAG epitope (DYKDDDDK, Biolegend, San Diego, CA, catalog# 637301) with 1:2 000 dilution overnight in the cold room, and the secondary antibody anti-rat IgG HRP (Cell Signaling Technology, Danvers, MA, catalog# 7077) with the same dilution at room temperature for 1 h. Premixed Pierce^TM^ 3,3 diaminobenzidine (DAB) substrate (Fisher Scientific) was used to detect the target proteins.

### RNA-seq

The quality of the RNA sample was assessed using a Nanodrop (Thermo Fisher Scientific) and an Agilent 5400 (Agilent Technologies, Santa Clara, CA, USA). Prokaryotic mRNA sequencing was performed using the NovaSeq PE150 platform (Illumina, San Diego, CA, USA) at the Novogen facility (Sacramento, CA, USA). The library was prepared by a Ribo-Zero protocol (250–300 bp insert strand-specific library with rRNA removal using NEB Ribo-Zero Magnetic Kit). Paired-end sequencing produced 150 bp reads to a depth of ~2 G output per sample. Sequences were mapped to a reference genome, *Fusobacterium nucleatum* ATCC 23726 (GenBank accession: CP028109) using a Bowtie2 pipeline adjusted for paired-end sequencing. Differential gene expression was analyzed using the DEseq2 pipeline in RStudio. The total mapping rates with respect to the annotated genome for *Fn* 23726 were >98%. The false discovery rate (FDR) was set to 5% and genes with a log_2_ FoldChange of >1 or <−1 and a *P*-value ≦0.05 were considered significant. All reported data are representative of three biological replicates.

### Statistical analysis

All statistical analyses and quantitative graphs were performed and prepared using R. Statistical significance was achieved through Student’s unpaired *t*-test: ****P < 0.000 1; ***P < 0.001; **P < 0.01; *P < 0.05.

## Supplementary information

Supplementary Information

## Data Availability

All study data are included in the article and/or Supplementary Information, the raw RNA-seq and Mass Spectrometry data were deposited in a public database: https://github.com/Pu-Ting/Fuso_PtaT.
