# Correction: Identification of a Fusobacterial RNA-binding protein involved in host small RNA-mediated growth inhibition

**DOI:** 10.1038/s41368-025-00389-1

**Published:** 2025-06-25

**Authors:** Pu-Ting Dong, Mengdi Yang, Jie Hu, Lujia Cen, Peng Zhou, Difei Xu, Peng Xiong, Jiahe Li, Xuesong He

**Affiliations:** 1 Department of Microbiology, The American Dental Association Forsyth Institute, Cambridge, MA USA; 2 Department of Bioengineering, Northeastern University, Boston, MA USA; 3 University of Science and Technology of China, Hefei, China; 4 Department of Biomedical Engineering, Suzhou Institute for Advanced Study, University of Science and Technology of China, Suzhou, China; 5 Department of Microbiology and Molecular Genetics, University of Texas McGovern Medical School, Houston, TX USA; 6 Department of Biomedical Engineering, College of Engineering and School of Medicine, University of Michigan, Ann Arbor, MI USA

**Keywords:** Cellular microbiology, Molecular medicine

Correction to: *International Journal of Oral Science* 10.1038/s41368-025-00378-4, published online 11 June 2025

Following publication of the original article^1^, it is reported that there is an error in the first author’s name, which should be corrected from Puting Dong to Pu-Ting Dong.

The original article^1^ has been updated.
