# Supplementary material for: Identification of a Fusobacterial RNA-binding protein involved in host small RNA-mediated growth inhibition
Source: Int J Oral Sci. 2025 Jun 11;17:48. doi: 10.1038/s41368-025-00378-4 (PMC12159189; doi:10.1038/s41368-025-00378-4)
Supplement: Supplementary file 1 — Supplementary Information [file 41368_2025_378_MOESM1_ESM.pdf]

Supplementary information for manuscript

Identification of a Fusobacterial RNA-binding protein involved in host small RNA-mediated growth inhibition

Pu-Ting Dong[1], Mengdi Yang[2,#], Jie Hu [3,4,#], Lujia Cen[1], Peng Zhou[5], Difei Xu[3,4], Peng Xiong[3,4,*], Jiahe Li[6,*], and Xuesong He[1,*]

[1]Department of Microbiology, The American Dental Association Forsyth Institute, Cambridge, MA 02142, USA.

[2]Department of Bioengineering, Northeastern University, Boston, MA 02115, USA.

[3]University of Science and Technology of China, Hefei 230026, China.

[4]Department of Biomedical Engineering, Suzhou Institute for Advanced Research, University of Science and Technology of China, Suzhou 215123, China.

[5]Department of Microbiology and Molecular Genetics, University of Texas McGovern Medical School, Houston, TX 77030, USA.

[6]Department of Biomedical Engineering, College of Engineering and School of Medicine, University of Michigan, Ann Arbor, MI 48109, USA.

[#]: These authors contribute equally.

[*]: Corresponding authors: Dr. Xuesong He (xhe@forsyth.org), Dr. Jiahe Li (jiaheli@umich.edu), Dr. Peng Xiong (xiongxp@ustc.edu.cn).

This document includes:

Figure S1-S10
Table S1 for PtaT sequence alignment is provided in a separate file
Table S2-S3

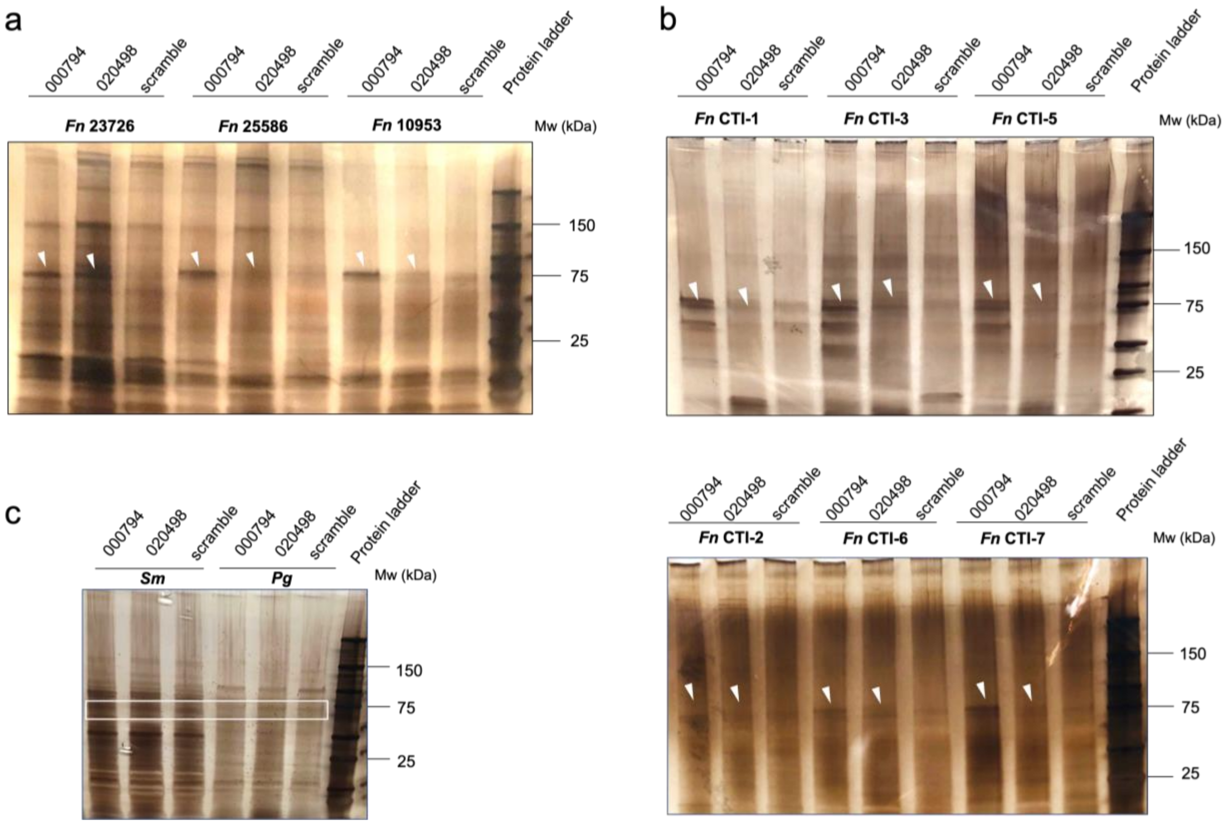

Supplementary Figure 1. Identification of PtaT by tsRNA-mediated affinity pulldown assay. a. Silver staining of denaturing SDS-PAGE gel for biotinylated tsRNA pulldown samples in three different ATCC *Fn* strains. Arrowheads indicate the gel bands, which were excised for protein identification by Mass Spectrometry analysis. **b.** Silver staining of denaturing SDS-PAGE gel for biotinylated tsRNA pulldown samples in six *Fn* clinical tumor isolates (CTIs). Arrowheads indicate the gel bands, which were excised for protein identification by Mass Spectrometry analysis. CTI-1: *F. nucleatum. ssp. animalis*; CTI-3: *F. nucleatum. ssp. animalis*; CTI-5: *F. nucleatum. ssp. animalis*; CTI-2: *F. nucleatum*, *ssp. nucleatum*; CTI-6: *F. nucleatum. ssp. polymorphum*; CTI-7: *F. nucleatum. ssp. vincentii.* **c.** Silver staining of denaturing SDS-PAGE gel for biotinylated tsRNA pulldown samples in *Streptococcus mitis* ATCC *6249* (*Sm*) and *Porphyromonas gingivalis* ATCC *33277* (*Pg*).

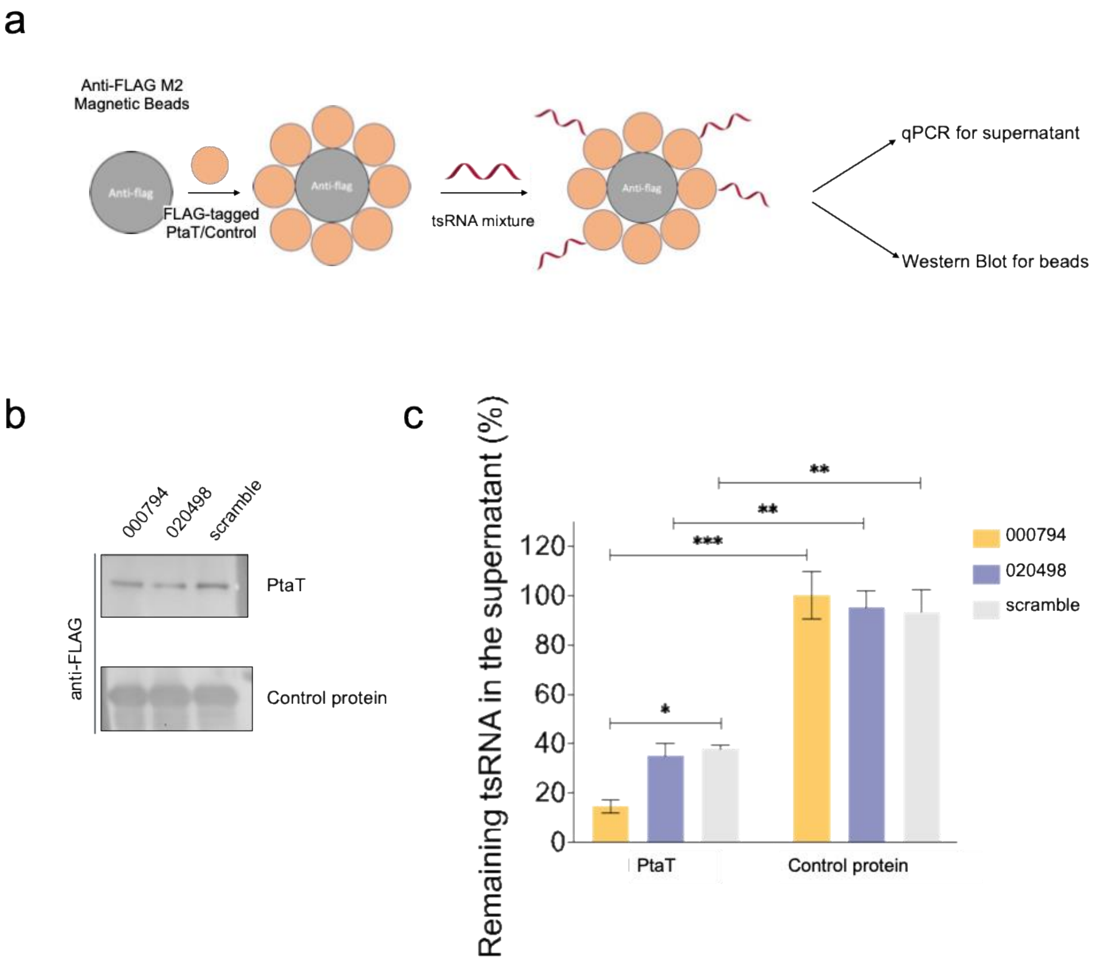

Supplementary Figure 2. Validation of the binding interaction between PtaT and tsRNA. a. Schematic of using anti-FLAG M2 magnetic beads and FLAG-tagged recombinant proteins to validate tsRNA binding *in vitro*. **b.** Western blotting of FLAG-tagged PtaT and STING (a negative control known to bind cyclic dinucleotides but not RNA oligos). **c.** Pull-down of naturally occurring tsRNA-000794, tsRNA-020498 or scramble control by purified FLAG-tagged PtaT and anti-FLAG antibody-conjugated magnetic beads. A FLAG-tagged irrelevant protein (STING) was used as a negative control for nonspecific binding. Unbound RNAs were quantified by stem-loop qPCR and normalized to the initial concentration. Results = Mean ± SEM (N=4) and are representative of two biological replicates. Statistical analyses were performed by the two-way ANOVA followed by Dunnett's Bonferroni multiple comparison tests. *$p < 0.05$, ** $p < 0.01$, *** $p < 0.001$.

Classification	Protein	Localization	Uniport	Groups
RNA metabolism	Polyribonucleotide nucleotidyltransferase	Cytoplasmic	D5RFI6	**tsRNA000794**
Metal ion transport	Heavy metal translocating P-type ATPase	Membrane	D5RD38	
	Uncharacterized		D5RA61	
Metabolic process	S-methyl-5-thioribose-1-phosphate isomerase	Membrane	D5RDB1	**tsDNA000794**
Metabolic process	Signal peptide peptidase SppA		D5REW1	
Transport	ABC transporter, substrate-binding protein		D5RBV3	
	Uncharacterized		D5RA61	
RNA metabolism	Ribonuclease J	Cytoplasmic	D5RD58	**piRNA_016792**
RNA metabolism	Polyribonucleotide nucleotidyltransferase	Cytoplasmic	D5RFI6	
	Uncharacterized		D5RA61	
RNA metabolism	Polyribonucleotide nucleotidyltransferase	Cytoplasmic	D5RFI6	**piRNA_006465**
RNA metabolism	Ribonuclease R	Cytoplasmic	D5RAL6	
	Uncharacterized		D5RA61	
Metabolic process	S-methyl-5-thioribose-1-phosphate isomerase		D5RDB1	**Beads only**

Supplementary Figure 3. Identification of RNA-binding proteins from *Fn* ATCC 23726 total lysate by RNA pulldown and Mass Spectrometry. P-type ATPase was highlighted by red in the table.

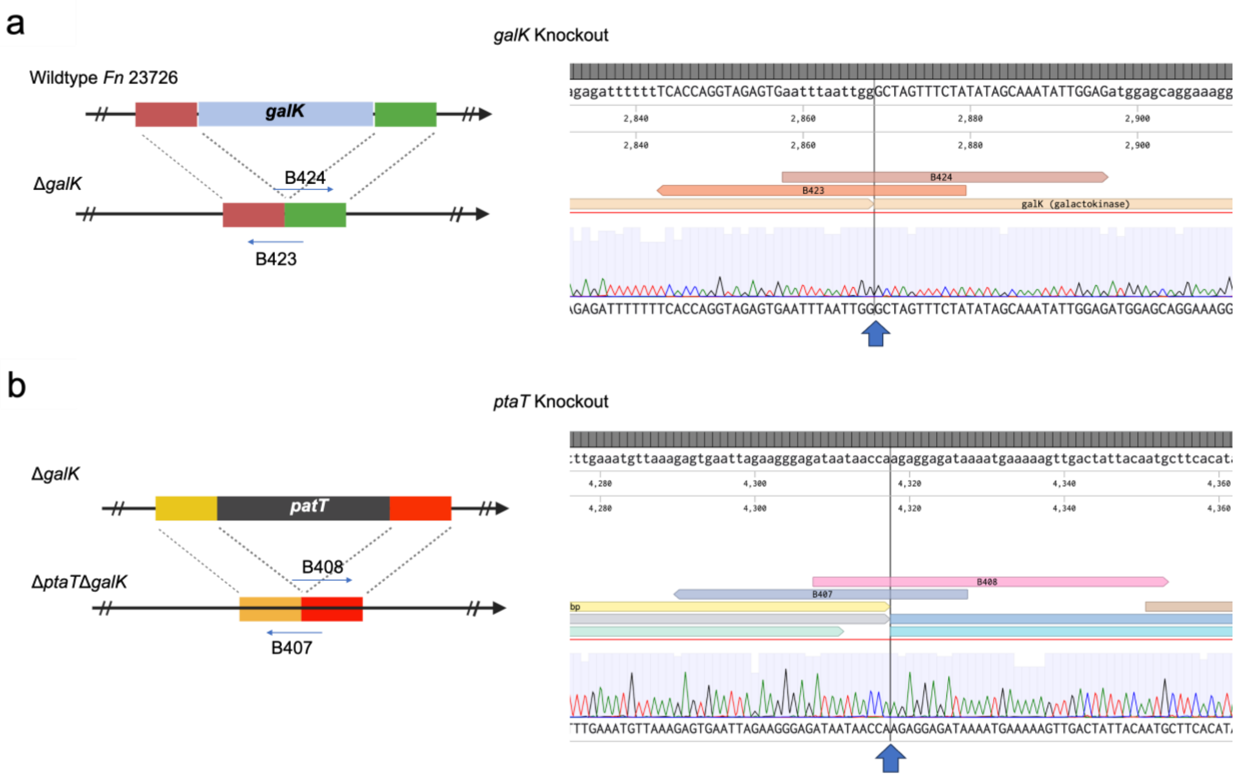

Supplementary Figure 4. A double crossover-mediated knockout of the full-length *ptaT* in *Fn* Δ*galK*. **a.** Generation of insertional mutagenesis via a double crossover at a 1kb central region of *galk* in *Fn* ATCC 23726. **b.** Double crossover-mediated complete knockout of the full-length *ptaT* in *Fn* ATCC 23726 Δ*galK*.

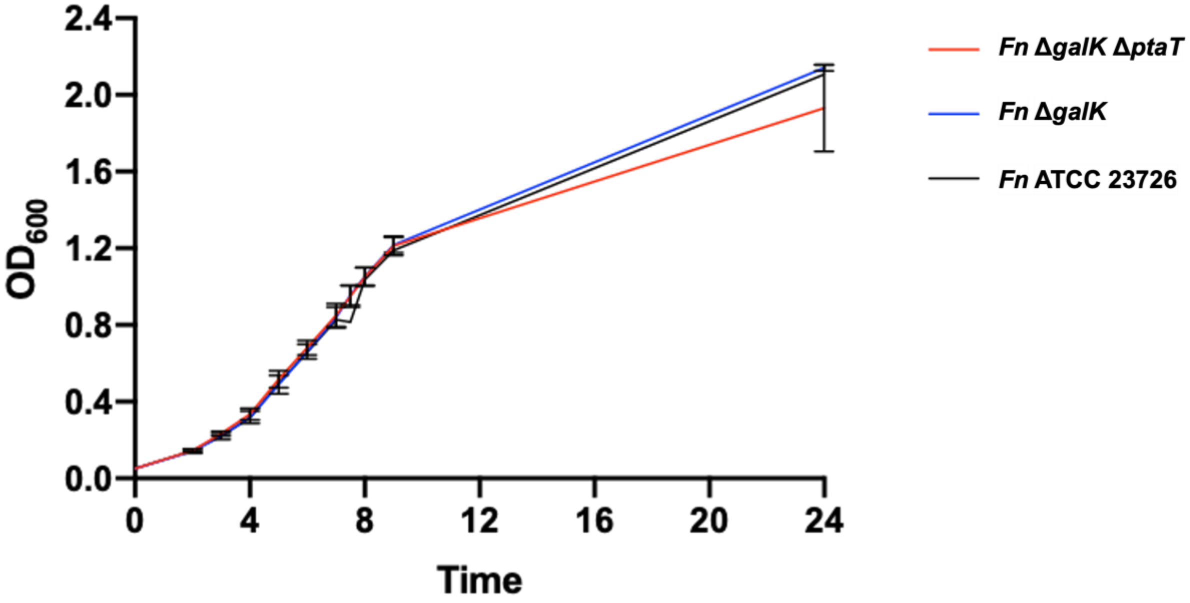

Supplementary Figure 5. **The time-course growth kinetic monitored by optical density at 600 nm (OD_{600}) from *Fn* ATCC 23726, *Fn* Δ*galK* and *Fn* Δ*galK* Δ*ptaT*. N = 3.**

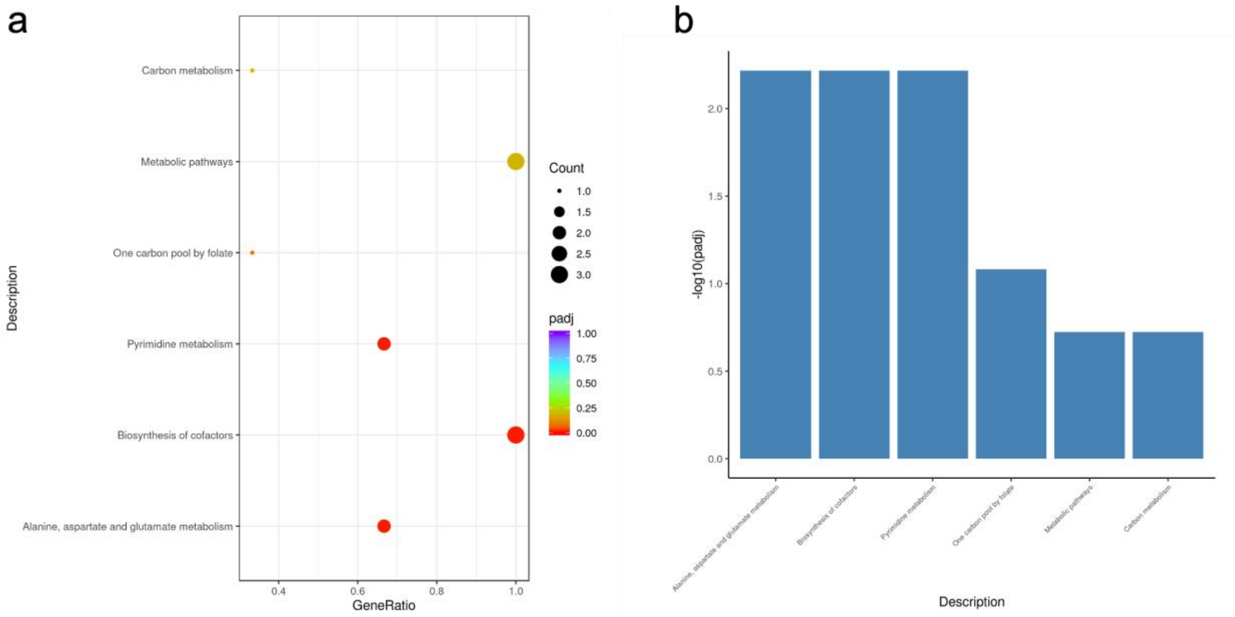

Supplementary Figure 6. Clusters of orthologous groups (COG, a) and quantification of differentially expressed genes (b) from stationary-phase *Fn* Δ*galK* Δ*ptaT* relative to *Fn* Δ*galK*.

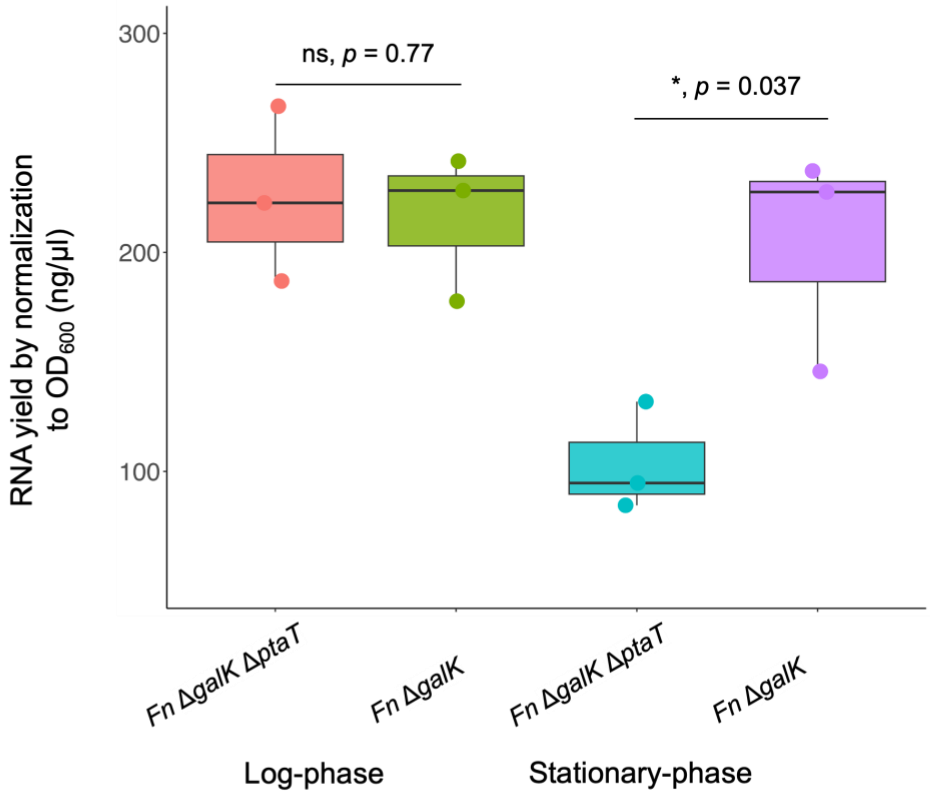

Supplementary Figure 7. The yield of total extracted RNA of both *Fn ΔgalK* and *Fn ΔgalK ΔptaT* from three biological replicates. Significant difference was determined through two-tailed unpaired Student's *t*-test.

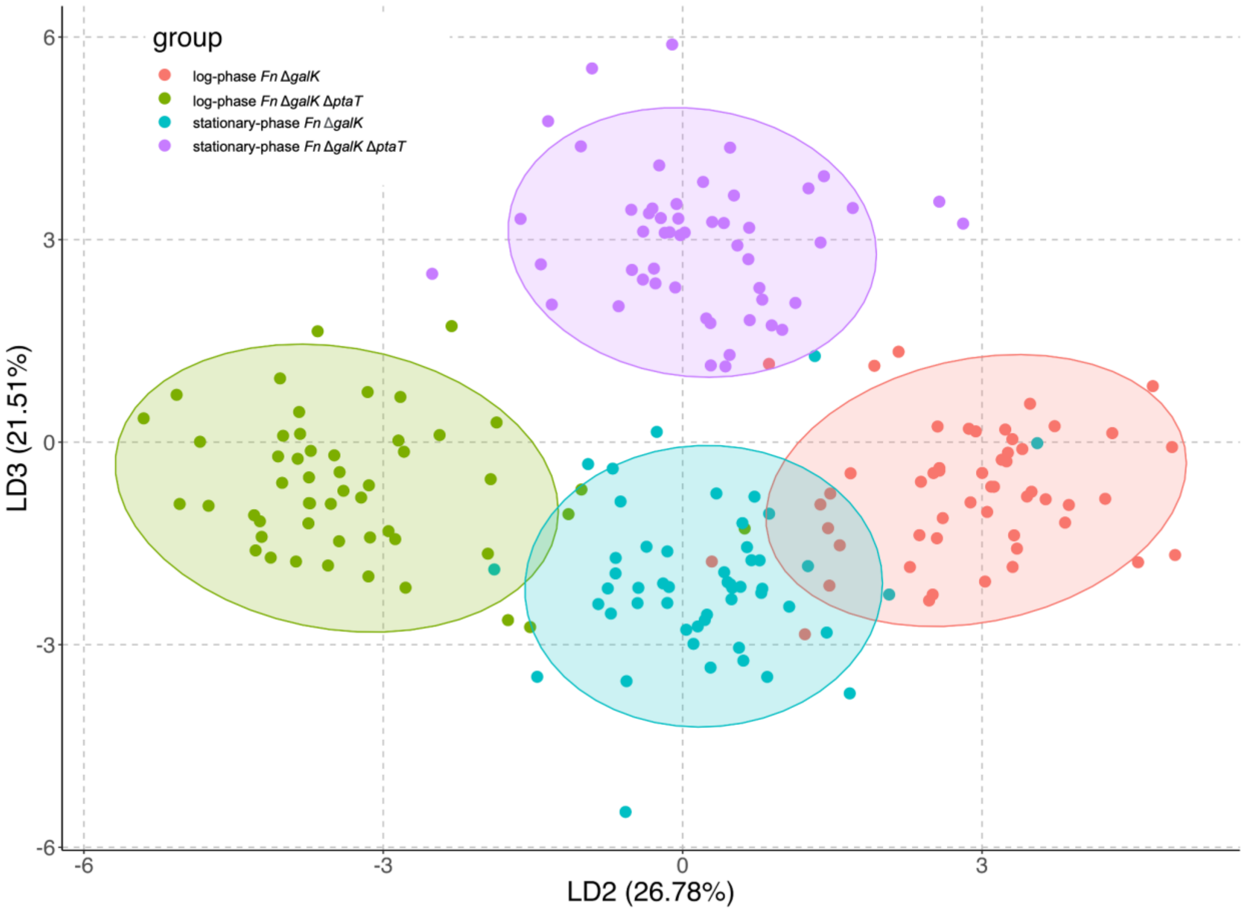

Supplementary Figure 8. LDA analysis (LD3 versus LD2) of 200 Raman spectra from log-phase and stationary-phase *Fn* Δ*galK* and *Fn* Δ*galK* Δ*ptaT*.

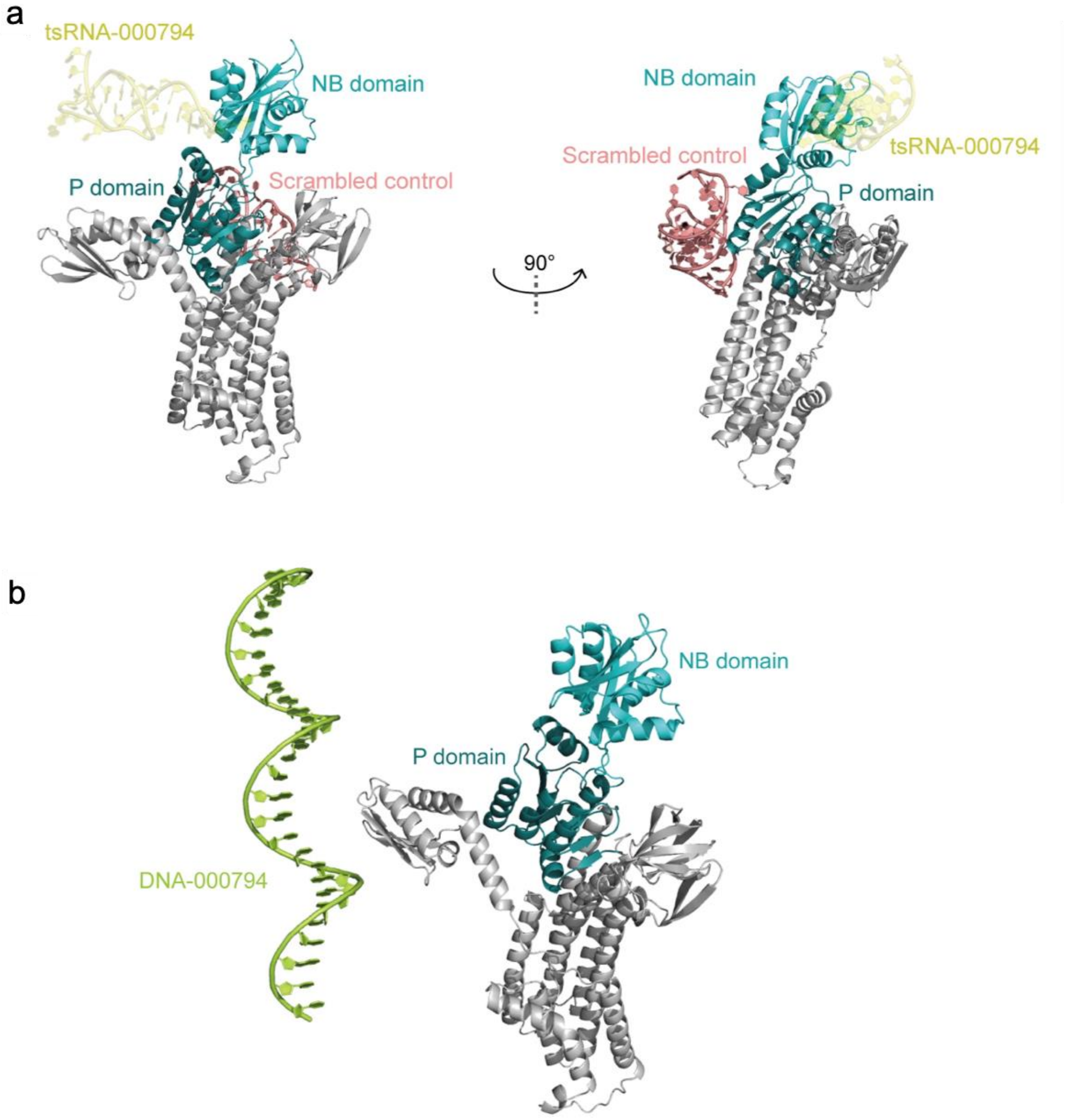

Supplementary Figure 9. Structural analysis of the predicted complex model between scrambled control (a), tsDNA-000794 (b) and PtaT by AlphaFold 3.

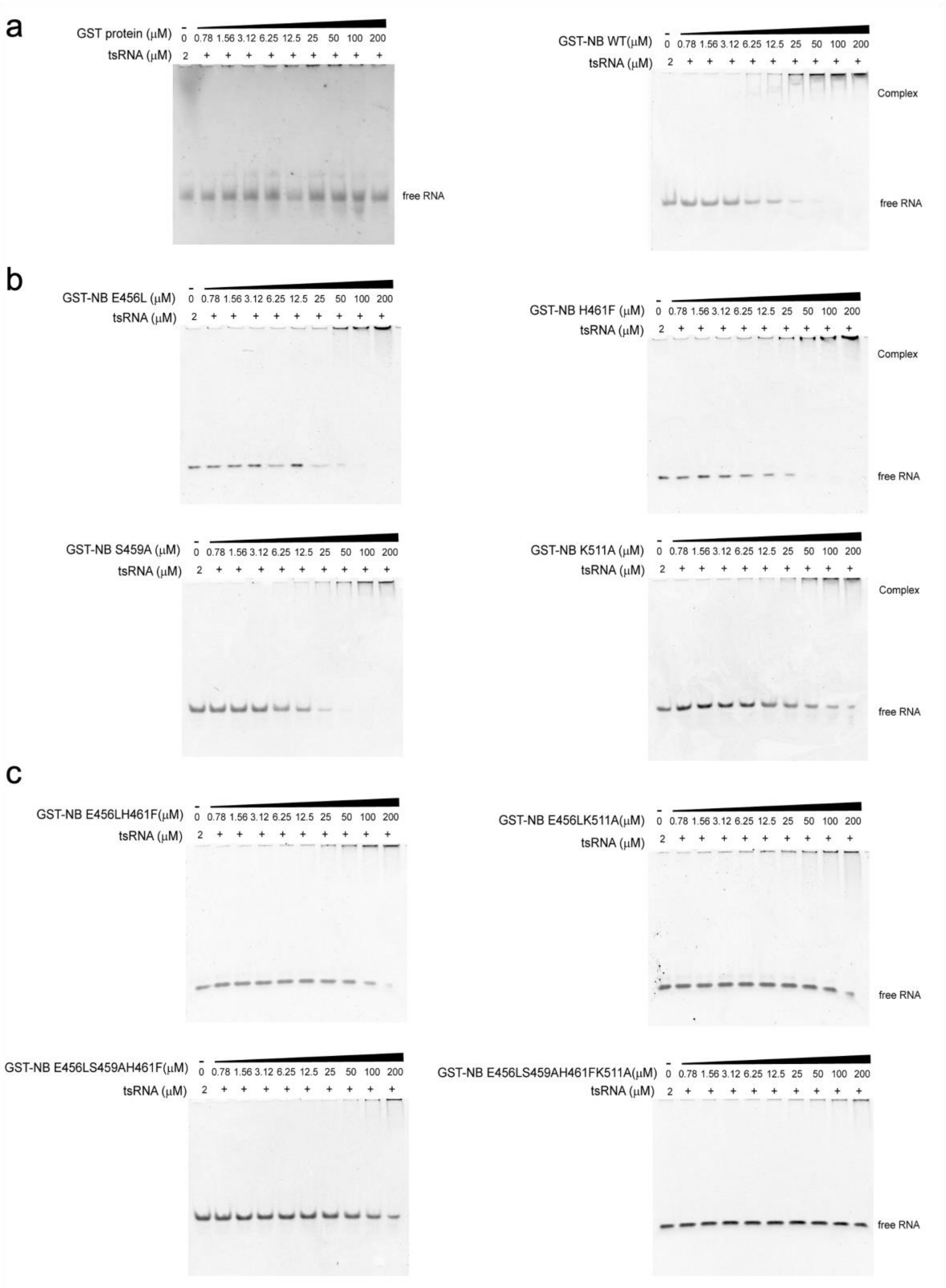

Supplementary Figure 10. EMSA results of alanine scanning mutations. a. GST negative control and wild-type control; **b.** Single-site mutations; **c**. Double and multi-sites mutations.

Table S2 Bacterial strains and plasmids used in this paper

Bacterial Species	Strains	Characteristics	Source
Porphyromonas gingivalis	ATCC 33277	WT	ATCC
Streptococcus mitis	ATCC 6249	WT	ATCC
F. nucleatum	ATCC 23726	*ssp. nucleatum* WT	ATCC
	ATCC 25586	*ssp. nucleatum* WT	ATCC
	ATCC 10953	*ssp. nucleatum* WT	ATCC
	Fn_ΔgalK	*galK* markerless deletion	this study
	Fn_ΔgalK ΔptaT	*ptaT* markerless deletion	this study
	CTI-1 (ssp. *animalis*)	clinical tumor isolate	Previous study[1]
	CTI-2 (ssp. *nucleatum*)	clinical tumor isolate	Previous study[1]
	CTI-3 (ssp. *animalis*)	clinical tumor isolate	Previous study[1]
	CTI-5 (ssp. *animalis*)	clinical tumor isolate	Previous study[1]
	CTI-6 (ssp. *polymorphum*)	clinical tumor isolate	Previous study[1]
	CTI-7 (ssp. *vincentii*)	clinical tumor isolate	Previous study[1]

	CTI-2 Δ*ptaT*	deletion of *ptaT* in CTI-2	this study
Escherichia coli	NEB5α		NEB
	Rosetta (DE3)		NEB
	C2987	cloning host	NEB
**Plasmids**	**Purpose**	**Characteristics**	**Source**
pSH200-PtaT	PtaT purification	amp	this study
pHS31_FLAG_*galK*	In-frame deletion of *galK*	*catP*	this study
pHS31_FLAG-*galK*-Δ*ptaT*	In-frame deletion of *ptaT*	*catP*	this study
pBCG02	*E. coli*/*Fusobacterium* shuttle vector	$cm^R/thia^R$	ref[2]
pBCG02-CTI2-1787updn	Derivative of pBCG02 lacking *repA* and ori_{Fn}, deletion plasmid of *ptaT*		this study

Table S3 Primers used for strain construction.

Primer Name	Sequence (5' to 3')	Description
B267	cgaaaacctgtacttccagggatccAAAAATGATAATTTACTCGCTT	Fwd primer to clone *ptaT* from *Fn* genome.
B268	tggtggtggtgctcgagtgcggccgcTTATCATTTGTCATCGTCGTCTTTGTAGTCATTAGTTTTTATATCATATTTTAATAAT	Rev primer to clone *ptaT* from *Fn* genome.
B406	tttaaaagcggccgcggtacGTTATTAAAAATTTTAAAATTATTCAAAGTCTTGGAAG	Fwd primer to clone 750bp upstream gene of *ptaT*
B407	tatctcctcttggttaTTATCTCCCTTCTAATTCACTC	Rev primer to clone 750bp upstream gene of *ptaT*
B408	ATAAtaaccaagaggagataaaATGAAAAAGTTGACTATTACAATG	Fwd primer to clone 750bp downstream gene of *ptaT*
B409	agggacactttttcactcgaAGTTACATATTTTTCATTAAATTGACAATAGTCAC	Rev primer to clone 750bp downstream gene of *ptaT*
B422	aaagaaaaactgccgggtacTGTTAATCCATTTGCTACTGTTATTGC	Fwd primer to clone 1kb upstream gene of *galK*
B423	tagaaactagcCCAATTAAATTCACTCTACCTGGTGA	Fwd primer to clone 1kb upstream gene of *galK*
B424	aatttaattggGCTAGTTTCTATATAGCAAATATTGGAG	Fwd primer to clone 1kb downstream gene of *galK*
B425	cggggatcgatcccgggtacCTCTCCAAGCTTTTAAAGTTTTATCTGC	Fwd primer to clone 1kb downstream gene of *galK*
B459	tttaaaagcggccgcggtacCAACTTGGAACTATTCAAGAACAAT	Fwd primer to verify *ptaT* deletion
B462	agggacactttttcactcgaTATTCGTTCCATAAAATTTTCCTTAATAATT	Rev primer to verify *ptaT* deletion

CTI2-1787upF	GAGTGTGATATGTATGGAAGCTACGAAAGATGAAAGATAAAACG	pBCG02-CTI2-1787updn
CTI2-1787upR	CCATAGCAAAATTTTCTTTTATCTCAATTTCAGATTTTAAAG	pBCG02-CTI2-1787updn
CTI2-1787dnF	CTTTAAAATCTGAAATTGAGATAAAAGAAAATTTTGCTATGG	pBCG02-CTI2-1787updn
CTI2-1787dnR	CGGAAATTTCACTAGTTCTAGGACGCTTGATTTTTCTTATATAG	pBCG02-CTI2-1787updn
Det-1787-F	GAAAGATGAAAGATAAAACG	Confirms ptaT deletion
Det-1787-R	GCTTGATTTTTCTTATATAG	Confirms ptaT deletion
pCWU6-F	GTCCTAGAACTAGTGAAATTTCCG	Removes repA and oriFn from pBCG02
pCWU6-R	GTAGCTTCCATACATATCACACTC	Removes repA and oriFn from pBCG02
**Beginning of sequencing primers for plasmids confirmation**		
S99	AACCCCTCAAGACCCGTTTA	T7 terminator Rev; sequencing pHS200
S101	TAATACGACTCACTATAGGG	T7 promoter Fwd; sequencing pHS200
S124	CCCAGTCACGACGTTGTAAAACG	M13
S133	CAGAAGATAATGTAAAAAGAGTTC	*ptaT* internal primer for *Fn* 23726
S136	CCACTTCGACTGCACTCCCGAC	pHS31 digestion site Fwd
S137	GTCCCTAGCGCCTACGGGGAAT	pHS31 digestion site Rev
S139	TTAGGACGGCAATCAATCAA	*CatP* Fwd on pHS31
S140	AAACGGCAAATGTGAAATCC	*CatP* Rev on pHS31
S150	AGGTGCTAATATTCCAGTTGCTC	Sequencing KO *ptat* strain

*The lower letters are the overlap region. Fwd, forward; Rev, reverse. Nucleotides with upper case indicate sequences complementary to the genome; nucleotides with lower case indicate sequences matched to digested plasmid pHS31 for genetic knockout. Underlined are primer sequences specific to the ends of the corresponding assembly.

References:

1 Abed, J. *et al.* Fap2 mediates *Fusobacterium nucleatum* colorectal adenocarcinoma enrichment by binding to tumor-expressed Gal-GalNAc. *Cell Host & Microbe* **20**, 215-225 (2016).

2 Peluso, E. A., Scheible, M., Ton-That, H. & Wu, C. Genetic manipulation and virulence assessment of *Fusobacterium nucleatum*. *Current Protocols in Microbiology* **57**, e104 (2020).

Table S1

```
CLUSTAL W (1.81) multiple sequence alignment

23726
MKNDNLLACEIVHRLRGRIRIKSKAFKYIGNPLKSEIEKQLLQVRYIENVEISLVTGTIL
CTI-2
MKNDNLLACEIVHRLRGRIRIKSKAFKYIGNPLKSEIEKQLLQVRYIENVEISLVTGTIL
25586
MKNDNLLACEIVHRLRGRIRIKSKAFKYIGNPLKSEIEKQLLQVRYIENVEISLVTGTIL
10953
MKNDNLLACEIVHRLRGRIRIKSRAFKYIGNSLKAQIEKQLLQVRYIENVEISLITGTIL
CTI-6
MKNDNLLACEIVHRLRGRIRIKSKAFKYIGNSLKSEIEKQLLQVRYIENVEISLITGTIL
CTI-7
MKNDNLLTCEIVHRLRGRIRIKSKAFKYVGNSLKLEIEKHLLQVRYIKSVEISLITGTIL
CTI-3
MKNDNLLTCEIVHRLRGRIRIKSKAFKYVGNSLKSEIEKQLLQVRYIKSVEISLITGTIL
CTI-1
MKNDNLLTCEIVHRLRGRIRIKSKAFKYVGNSLKSEIEKQLLQVRYIKSVEISLITGTIL
CTI-5
MKNDNLLTCEIVHRLRGRIRIKSKAFKYVGNSLKSEIEKQLLQVRYIKSVEISLITGTIL

*******:***************.****:**.** :***:*******:.*****:*****

23726
IYFEDVSLSDQNLISLIQNTLNSHIFEICKNEKVEKSSKYIIERKLQEESPKEIMKKIVT
CTI-2
IYFEDVSLSDQNLISLIQNTLNSHIFEICKNEKVEKSSKYIIERKLQEESPKEIMKKIVT
25586
IYFEDVSLSDQNLISLIQNTLNSHIFEICKNEKVEKSSKYIIERKLQEESPKEIMKKIVT
10953
IYFEDVSLSDQNLISLIQNTLNSHIFEICKNEKIEKSSKYVIERKLQEESPKEIMKKILT
CTI-6
IYFEDVSLSDQNLISLIQNTLNSHIFEICKNEKIEKSSKYVIERKLQEESPKEIMKKILT
CTI-7
IYFEDVSLSDQNLINLIQNTLNSHIFEICKNEKIEKSSKYVIERKLQEESPKEIVKKIIA
CTI-3
IYFEDVSLSDQNLINLIQNTLNSHIFEICKNEKVEKSSKYVIERKLQEESPKEIVKKIIA
CTI-1
IYFEDVSLSDQNLINLIQNTLNSHIFEICKNEKVEKSSKYVIERKLQEESPKEIVKKIIA
CTI-5
IYFEDVSLSDQNLINLIQNTLNSHIFEICKNEKVEKSSKYVIERKLQEESPKEIVKKIIA

**************.******************:******:*************:***::

23726
TAGLLGYNLFFKSKSTVALTGIRRFLNYNTLSTLALAMPVLKNGINSLIKNKRPNADTLS
CTI-2
TAGLLGYNLFFKSKSTVALTGIRRFLNYNTLSTLALAMPVLKNGINSLIKNKRPNADTLS
25586
TAGLLGYNLFFKSKSTVALTGIRRFLNYNTLSTLALAMPVLKNGINSLIKNKRPNADTLS
```

10953
TAGLLGYNLFFKSKNTAALTGIRRFLNYNTLSTLALAMPVLKNGINSLVKNKRPNADTLS
CTI-6
TAGLLGYNLFFKSKSTVALTGIRRFLNYNTLATLALAMPVLKNGINSLVKNKRPNADTLS
CTI-7
TAGLLGYNLFFKSKSTVALTGIRKFLNYNTLSTLALAMPVLKNGINSLIKNKRPNADTLS
CTI-3
TAGLLGYNLFFKPKSTVALTGIRRFLNYNTFSTLALAMPVLKNGVNSLIKNKRPNADTLS
CTI-1
TAGLLGYNLFFKSKSPVALTGIRRFLNYNTLSTLALAMPVLKNGVNSLIKNKRPNADTLS
CTI-5
TAGLLGYNLFFKPKSTVALTGIRRFLNYNTLSTLALAMPVLKNGVNSLIKNKRPNADTLS

************.*...******.******::************:***:***********

23726
SSAIISSILLGKESAALTIMFLEEVSELLTVYTMEKTRGAIKDMLSVGENYVWKEISEDN
CTI-2
SSAIISSILLGKESAALTIMFLEEVSELLTVYTMEKTRGAIKDMLSVGENYVWKEISEDN
25586
SSAIISSILLGKESAALTIMFLEEVSELLTVYTMEKTRGAIKDMLSVGENYVWKEISEDN
10953
SSAIISSILLGKESAALTIMFLEEVSELLTVYTMEKTRGAIKDMLSVGENYVWKEISEDN
CTI-6
SSAIISSILLGKESAALTIMFLEEVSELLTVYTMEKTRGAIKDMLSVGENYVWKEISEDN
CTI-7
SSAIISSILLGKESAALTIMFLEEVSELLTVYTMEKTRGAIKDMLSVGENYVWKEISEDN
CTI-3
SSAIISSILLGKESAALTIMFLEEVSELLTVYTMEKTRGAIKDMLSVGENYVWKEISEDN
CTI-1
SSAIISSILLGKESAALTIMFLEEVSELLTVYTMEKTRGAIKDMLSVGENYVWKEISEDN
CTI-5
SSAIISSILLGKESAALTIMFLEEVSELLTVYTMEKTRGAIKDMLSVGENYVWKEISEDN

****

23726
VKRVPIEEIQKDDIIVVQTGEKISVDGKIIRGEALIDQSSITGEYMPIKKSEGEEVYAGT
CTI-2
VKRVPIEEIQKDDIIVVQTGEKISVDGKIIRGEALIDQSSITGEYMPIKKSEGEEVYAGT
25586
VKRVPIEEIQKDDIIVVQTGEKISVDGKIIRGEALIDQSSITGEYMPIKKSEGEEVYAGT
10953
VKRVPIEEIQKDDIIVVQTGEKISVDGKIIRGEALIDQSSITGEYMPIKKSIGEDVYAGT
CTI-6
VKRVPIEEIQKDDIIVVQTGEKISVDGKIIRGEALIDQSSITGEYMPIKKSIGEDVYAGT
CTI-7
VKRVPIEEIKKDDIIVVQTGEKISVDGKIIRGEALIDQSSITGEYMPIKKSVEDDVYAGT
CTI-3
VKRVPIEEIQKDDIIVVQTGEKISVDGKIIKGEALIDQSSITGEYMPIKKSKGDDVYAGT

```
CTI-1
VKRVPIEEIQKDDIIVVQTGEKISVDGKIIKGEALIDQSSITGEYMPIKKSKGDDVYAGT
CTI-5
VKRVPIEEIQKDDIIVVQTGEKISVDGKIIKGEALIDQSSITGEYMPIKKSKGDDVYAGT

*********:********************.********************  ::*****

23726
IIKNGNISIIAEKVGDDRTVSRIIKLVEDANSNKADIQNYADTFSAQLIPLNFILAGIVY
CTI-2
IIKNGNISIIAEKVGDDRTVSRIIKLVEDANSNKADIQNYADTFSAQLIPLNFILAGIVY
25586
IIKNGNISIIAEKVGDDRTVSRIIKLVEDANSNKADIQNYADTFSAQLIPLNFILAGIVY
10953
IVKNGNISIIAEKVGDDRTVSRIIKLVEDANSNKADIQNYADTFSAQLIPLNFILAGIVY
CTI-6
IVKNGNISIIAEKVGDDRTVSRIIKLVEDANSNKADIQNYADTFSAQLIPLNFILAGIVY
CTI-7
IVKNGNISIIAEKVGDDRTVSRIIKLVEDANSNKADIQNYADTFSAQLIP----------
CTI-3
IVKNGNISIIAEKVGDDRTVSRIIKLVEDANSNKADIQNYADTFSAQLIPLNFILAGIVY
CTI-1
IVKNGNISIIAEKVGDDRTVSRIIKLVEDANSNKADIQNYADTFSAQLIPLNFILAGIVY
CTI-5
IVKNGNISIIAEKVGDDRTVSRIIKLVEDANSNKADIQNYADTFSAQLIPLNFILAGIVY
                *:************************************************

23726
ASTRSITKAMSMLVIDYSCGIRLSTAVAFSAAINTAAKNGILVKGSNFIEELSKSETVIF
CTI-2
ASTRSITKAMSMLVIDYSCGIRLSTAVAFSAAINTAAKNGILVKGSNFIEELSKSETVIF
25586
ASTRSITKAMSMLVIDYSCGIRLSTAVAFSAAINTAAKNGILVKGSNFIEELSKSETVIF
10953
ASTRNITKAMSMLVIDYSCGIRLSTAVAFSAAINTAAKNGILVKGSNFIEELSKAETVIF
CTI-6
ASTRNITKAMSMLVIDYSCGIRLSTAVAFSAAINTAAKNGILVKGSNFIEELSKAETVIF
CTI-7
------------------------------------------------------------
CTI-3
ASTRSLTKAMSMLVIDYSCGIRLSTAVAFSAAINTAAKNGILVKGSNFIEELSKAETVIF
CTI-1
ASTRSLTKAMSMLVIDYSCGIRLSTAVAFSAAINTAAKNGILVKGSNFIEELSKAETVIF
CTI-5
ASTRSLTKAMSMLVIDYSCGIRLSTAVAFSAAINTAAKNGILVKGSNFIEELSKAETVIF


23726
DKTGTITEGKPKVQSIEVFDNNMSENEMIGLAGAAEEQSSHPLATAIMSEIKDRGIEIPK
CTI-2
```

DKTGTITEGKPKVQSIEVFDNNMSENEMIGLAGAAEEQSSHPLATAIMSEIKDRGIEIPK
25586
DKTGTITEGKPKVQSIEVFDNNMSENEMIGLAGAAEEQSSHPLATAIMSEIKDRGIEIPK
10953
DKTGTITEGKPKVQSIEVFDNSISENEMIGLAGAAEEQSSHPLAIAIMSEIKDRGIEIPK
CTI-6
DKTGTITEGKPKVQSIEVFDNSISENEMIGLAGAAEEQSSHPLATAIMSEIKDRGIEIPK
CTI-7

CTI-3
DKTGTITEGKPKVQSIEIFDNSISENEMIGLAGAAEEQSSHPLATAIMSEIKDRGIEIPK
CTI-1
DKTGTITEGKPKVQSIEIFDNSISENEMIGLAGAAEEQSSHPLATAIMSEIKDRGIEIPK
CTI-5
DKTGTITEGKPKVQSIEIFDNSISENEMIGLAGAAEEQSSHPLATAIMSEIKDRGIEIPK

23726
HNKIKTVVSRGVETKIGKGKEAKIIRVGSKKYMLENNIDLTLATEAERGIISRSEIGLYV
CTI-2
HNKIKTVVSRGVETKIGKGKEAKIIRVGSKKYMLENNIDLTLATEAERGIISRSEIGLYV
25586
HNKIKTVVSRGVETKIGKGKEAKIIRVGSKKYMLENNIDLTLATEAERGIISRSEIGLYV
10953
HNKIKTVVSRGVETKVGKGKEAKTIRVGSKKYMLENNIDLTLATEAERGIISRSEIGLYV
CTI-6
HNKIKTVVSRGVETKVGKGKEAKTIRVGSKKYMLENNIDLTLATEAERGIISRSEIGLYV
CTI-7

CTI-3
HNKIKTVVSRGVETKIGKGKDAITIRVGSKKYMLENNVDLTLATNAERGIISRGEIGLYV
CTI-1
HNKIKTVVSRGVETKIGKGKDAITIRVGSKKYMLENNVDLTLATNAERGIISRGEIGLYV
CTI-5
HNKIKTVVSRGVETKIGKGKDAITIRVGSKKYMLENNVDLTLATNAERGIISRGEIGLYV

23726
AQDEKIIGLIGVSDPPRENIKKAINRLRNYGVDDIVLLTGDLRQQAETIASRMSIDRYES
CTI-2
AQDEKIIGLIGVSDPPRENIKKAINRLRNYGVDDIVLLTGDLRQQAETIASRMSIDRYES
25586
AQDEKIIGLIGVSDPPRENIKKAINRLRNYGVDDIVLLTGDLRQQAETIASRMSIDRYES
10953
SQDEKIIGLIGVSDPPRENIKKAINRLRNYGVDDIVLLTGDLRQQAETIASRMSIDRYES
CTI-6
AQDEKIIGLIGVSDPPRENIKKAINRLRNYGVDDIVLLTGDLRQQAETIASRMSIDRYES
CTI-7

CTI-3

AQNEKIIGLIGVSDPPRENIKKAINRLRNYGVDDIVLLTGDLRQQAETIASRMSIDRYES
CTI-1
AQNEKIIGLIGVSDPPRENIKKAINRLRNYGVDDIVLLTGDLRQQAETIASRMSIDRYES
CTI-5
AQNEKIIGLIGVSDPPRENIKKAINRLRNYGVDDIVLLTGDLRQQAETIASRMSIDRYES

23726
ELLPEDKAKNILKFQSKGSNVIMIGDGVNDAPALSYANVGVALGSTRTDVAMEAADITIT
CTI-2
ELLPEDKAKNILKFQSKGSNVIMIGDGVNDAPALSYANVGVALGSTRTDVAMEAADITIT
25586
ELLPEDKAKNILKFQSKGSNVIMIGDGVNDAPALSYANVGVALGSTRTDVAMEAADITIT
10953
ELLPEDKAKNILKFQSKGSNVIMIGDGVNDAPALSYANVGVALGSTRTDVAMEAADITIT
CTI-6
ELLPEDKAKNILKFQSKGSNVIMIGDGVNDAPALSYANVGVALGSTRTDVAMEAADITIT
CTI-7

CTI-3
ELLPEDKAKNILKFQSKGSNVIMIGDGVNDAPALSYANVGVALGSTRTDVAMEAADITIK
CTI-1
ELLPEDKAKNILKFQSKGSNVIMIGDGVNDAPALSYANVGVALGSTRTDVAMEAADITIT
CTI-5
ELLPEDKAKNILKFQSKGSNVIMIGDGVNDAPALSYANVGVALGSTRTDVAMEAADITIT

23726
QDNPLLVPGVIGLSKNTVKTIKENFAMVIGLNTFALVLGATGILAPIYASVLHNSTTILV
CTI-2
QDNPLLVPGVIGLSKNTVKTIKENFAMVIGLNTFALVLGATGILAPIYASVLHNSTTILV
25586
QDNPLLVPGVIGLSKNTVKTIKENFAMVIGLNTFALVLGATGILAPIYASVLHNSTTILV
10953
QDNPLLVPGVIGLSKNTVKTIKENFAMVIGLNTFALVLGATGILAPIYASVLHNSTTILV
CTI-6
QDNPLLVPGVIGLSKSTVKTIKENFAMVIGLNTFALVLGATGILAPIYASVLHNSTTILV
CTI-7

CTI-3
QDNPLLVPGVIGLSKNTVKTIKENFAMVIGLNTFALVLGATGILAPIYASVLHNSTTILV
CTI-1
QDNPLLVPGVIGLSKNTVKTIKENFAMVIGLNTFALVLGATGILAPIYASVLHNSTTILV
CTI-5
QDNPLLVPGVIGLSKNTVKTIKENFAMVIGLNTFALVLGATGILAPIYASVLHNSTTILV

23726 VMNSLKLLKYDIKTN
CTI-2 VMNSLKLLKYDIKTN
25586 VLNSLKLLKYDIKTN

10953	VLNSLKLLKYDIKTN
CTI-6	VMNSLKLLKYDIKTN
CTI-7	---------------
CTI-3	VMNSLKLLKYDIKTN
CTI-1	VMNSLKLLKYDIKTN
CTI-5	VMNSLKLLKYDIKTN
